# Imaging hole transport at catalyst-coated MIS photoanodes for water splitting under high-intensity illumination

**DOI:** 10.1039/d5sc08974c

**Published:** 2026-02-20

**Authors:** Kanokwan Klahan, Bertrand Goudeau, Patrick Garrigue, Véronique Lapeyre, Lionel Santinacci, Mana Toma, Pichaya Pattanasattayavong, Gabriel Loget

**Affiliations:** a University of Bordeaux, Bordeaux INP, ISM, UMR CNRS 5255 Pessac 33607 France gabriel.loget@cnrs.fr; b Department of Materials Science and Engineering, School of Molecular Science and Engineering, Vidyasirimedhi Institute of Science and Technology (VISTEC) Rayong 21210 Thailand pichaya.p@vistec.ac.th; c Aix-Marseille Univ., CNRS, CINaM Marseille France; d Department of Electrical and Electronic Engineering, School of Engineering, Institute of Science Tokyo Nagatsuta-cho 4259, Midori-ku Yokohama 226-8501 Japan

## Abstract

Metal–insulator–semiconductor (MIS) photoanodes are increasingly employed for solar water splitting due to their high performance. Here, we introduce a photoelectrochemical (PEC) mapping system that utilizes the scanning light beam of a confocal microscope, focused onto the surface of a photoanode. This approach enables submicrometric spatial resolution, which we employ to study photocurrent generation in MIS photoanodes constructed from n-type Si (n-Si) coated with oxygen evolution reaction (OER)-active Ni micropatterns. Our study highlights several key features. First, minority carrier (hole) transport beneath the uncoated SiO_*x*_ surface is influenced by the presence of electrolyte at the interface. Second, the main photocurrent contribution arises from illumination of Ni-free regions, even mm away from the Ni active sites. Third, the hole collection at the catalyst becomes significantly limited under high-intensity illumination regimes. These results are rationalized, allowing a general description of the transport of photogenerated holes in these MIS photoanodes. Furthermore, PEC mapping directly reveals that the issue of limited hole collection at high illumination intensities can be mitigated by shortening the spacing between catalyst islands. These findings offer key insights for designing more efficient PEC-based solar fuel systems that perform efficiently under high light intensities, such as concentrated sunlight.

## Abstract 2

Metal–insulator–semiconductor (MIS) photoanodes, increasingly employed for solar water splitting, are mapped photoelectrochemically, providing insights on charge collection and transfer under different illumination regimes.

## Graphical abstract

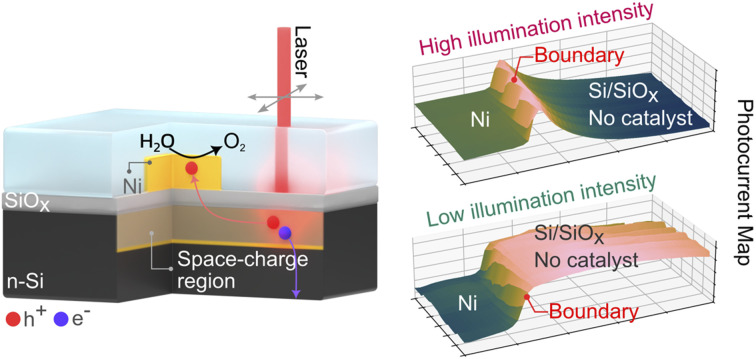

## Introduction

The conversion of sunlight energy into solar fuels has been identified as a promising solution for energy and climate sustainability.^1–3^ Toward this goal, the development of efficient and stable photoelectrodes remains crucial to the practical applications of photoelectrochemical (PEC) solar energy technologies. Photoelectrodes, key elements of PEC systems, are based on a (n- or p-type) semiconductor absorber, usually coated with protective and/or catalytic coatings. Most photoelectrodes contain micro/nanostructures on their surface, incidentally or intentionally introduced [*e.g.*, cracks,^4^ crystal facets,^5^ grain boundaries,^6^ catalytic nanoparticles (NPs),^7^ or single-atom catalysts,^8^ among others]. Surface composition often alters the PEC performance, which strongly depends on the photoactive junction, as well as solid/solid and solid/liquid charge transfer mechanisms occurring inside or through the outer surface of the photoelectrode.^9,10^ Thus, access to the local PEC properties at the micro/nano-structural level is of major interest and has already led to important discoveries and improvements for solar energy conversion.^4,11,12^ In general, research interest has focused on elucidating the local kinetics of charge transfer and product generation using PEC mapping devices.^11,13,14^ Besides, PEC mapping can also be utilized for the combinatorial screening of photo (electro)catalysts.^15,16^ Several setups and methods have been employed, including scanning electrochemical microscopy (SECM,^17^*i.e.*, local product detection under global or local illumination),^18–22^ scanning photocurrent microscopy (SPCM, *i.e.*, photocurrent measurement under local illumination),^23–26^ photoconductive atomic force microscopy,^27^ and photoinduced electrochemiluminescence (PECL) microscopy (*i.e.*, observation of luminescence generated *via* interfacial charge transfer of minority carriers).^28–30^

Si-based photoelectrodes have been widely utilized and studied as model systems for PEC water splitting.^31–33^ Generally, the Si surface is coupled with a protection and/or a catalyst coating to achieve high PEC performance for both photoanodes^34^ and photocathodes.^35^ Among Si-based photoelectrodes, metal–insulator–semiconductor (MIS) photoanodes are increasingly popular due to their high performance and reliability for solar water splitting. Such photoanodes, thoroughly characterized in previous publications,^32,36–39^ consist of a moderately doped (∼10^15^ at. per cm^3^) n-type Si (n-Si) light absorber, a thin tunnel layer (in our case, ∼2 nm-thick SiO_*x*_), covered by a transition metal catalyst. The metal coatings are not limited to planar conformal films but can also be inhomogeneous, such as a patterned layer,^40^ transition metal micropatches/microdots,^18^ and NPs.^41^ Inhomogeneity can also be created during operation due to the evolution of the interface and subsurface. For instance, Si-based photoanodes initially covered with a planar Ni thin film were found to evolve considerably during PEC measurements, which promoted an increase in performance.^42,43^ Attempts to understand and visualize the process occurring locally on Si photoelectrodes have been made *via* several techniques. Talin *et al.* studied the local reaction and photocurrent on MIS Si photocathodes coated with Pt patches using SECM and SPCM.^44^ They suggested that the photocurrent was only generated when the edges of the Pt patches and the surrounding Si surface were illuminated while SECM revealed the reaction occurring on the Pt charge collector. n-Si photoanodes modified with Ni micropatches were also investigated by SECM, showing the product (O_2_) generation predominantly at the Ni surface.^18^ Recently, our group has employed PECL microscopy for the direct observation of local charge transfer at micropatterned^28^ and Ni NPs-covered n-Si photoanodes.^29^ It was found that photogenerated solid/liquid charge transfer occurs efficiently and regioselectively onto the transition metal patches and NPs, down to a size of ∼50 nm. However, up to now, many uncertainties remain regarding the interactions between these different regions and their contribution to the overall current. In addition, the utilization of highly concentrated sunlight for solar-assisted fuel and chemical productions has been emerging recently.^3^ Photoelectrodes (*e.g.*, Fe_2_O_3_,^45–47^ BiVO_4_,^47^ np^+^-Si/GaN,^48^ or Si^49^) and photovoltaics (PV) coupled systems^50^ were tested under concentrated sunlight (≥10 suns). Under these conditions, the system may behave differently than under normal 1-sun illumination. Thus, the investigation of the local photoelectrode activities and properties under high-power illumination is of great importance for future developments in this field.

**Fig. 1:**
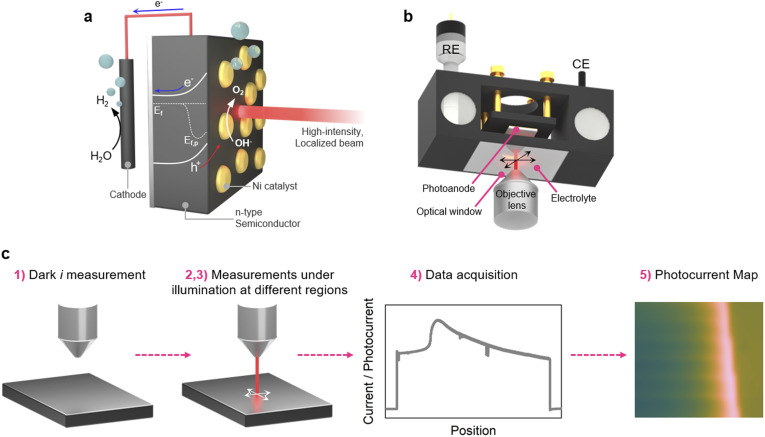
(a) Scheme displaying the photoelectrochemical system consisting of a photoanode coated with a catalyst layer under a high-intensity, localized laser beam, and a cathode. *E*_f_ and *E*_f,p_ is the Fermi level and the quasi-Fermi level for holes under illumination, respectively. (b) Scheme of the PEC cell and the setup for the PEC mapping based on an inverted confocal microscope, viewed from the bottom. (c) Scheme showing the main procedure and the five steps employed for PEC mapping. REF and CE stand for the reference and counter electrode, respectively.

In this work, we present a PEC mapping system based on a scanning light beam, produced by a confocal microscope, which is focused onto a photoanode surface (Fig. 1a). We demonstrate that this system allows a submicrometric resolution, and we use it to investigate photocurrent generation at MIS n-Si-based photoanodes coated with micropatterned Ni layers. This study shows that holes (h^+^) photogenerated in n-Si can be directed to the Ni surface over a mm distance and consumed for the oxygen evolution reaction (OER) at the Ni/electrolyte interface. Furthermore, our results reveal several important characteristics, namely: (i) hole transport beneath the uncoated SiO_*x*_ surface depends on the presence of electrolyte at the interface, (ii) the greater contribution of photocurrent originates from illumination of areas without Ni even mm away from the Ni active sites, and (iii) a significant limitation in hole collection at the catalyst occurs under high-intensity illumination conditions.

## Results and discussion

### Description of the PEC mapping system

Schemes of the PEC cell and the experimental method are shown in Fig. 1b and c (full details are given in the Methods section in the SI). In short, the PEC mapping system is based on the combination of an inverted confocal microscope with a PEC cell. The objective lens is facing upward, allowing illumination of the photoanode (in contact with the electrolyte and under potential control) at a precise location with a light spot (wavelength: 633 nm). While the stage is fixed during the PEC mapping, the scanning of the laser beam across the surface is precisely controlled by the oscillating mirrors of the confocal microscope. As shown in Fig. 1c, the principle of this PEC mapping system can be described as follow: (step 1) a fixed potential is applied to the sample and the current is recorded in dark condition to collect the dark background current (*i*_d_); (step 2) the laser locally illuminates the photoelectrode generating charge carriers and the overall current (*i*) including the photocurrent (*i* = *i*_d_ + *i*_ph_) is measured; (step 3) the laser is aligned on the next assigned areas and step 2 is repeated; (step 4) the current is acquired; and (step 5) a *i*_ph_ map or profile is generated after data collection on all the desired area. An important aspect that should be noted is that the *i* value measured corresponds to the process occurring on the entire surface and not only from the illuminated area. This is particularly important here since Si has a large carrier diffusion length (*vide infra*), meaning that locally photogenerated minority carriers (h^+^ in the case of n-Si used here) can promote faradaic reactions far from the light spot.^13,51–53^

Although this system will be mainly employed for PEC mapping in potentiostatic conditions, to comprehend the photoanode's reactivity, we started by studying the voltametric response of a uniformly coated photoanode (Section 2 in the SI). We first investigated Si photoanodes uniformly coated with a Ni thin film (40 nm, ∼50% transmittance at 633 nm) under localized illumination to establish the electrochemical response of the catalyst layer. Cyclic voltammograms recorded on the Ni-covered surface show a pronounced anodic redox feature between ∼0.6 and 0.9 V *vs.* Hg/HgO, which we attribute to the quasi-reversible Ni^II^/Ni^III^ [Ni(OH)_2_/NiOOH] transformation, in agreement with previous reports.^36,54^ Under illumination, the anodic current increases markedly, indicating participation of photogenerated holes in the oxidation processes, whereas bare n-Si/SiO_*x*_ electrodes exhibit negligible photoresponse under identical conditions (Fig. S1). Importantly, at potentials positive of the Ni redox transition, the anodic photocurrent persists beyond the redox peak, consistent with the onset of catalytic oxygen evolution following the formation of NiOOH. Since anodic photocurrents in Ni-based systems can originate from both Ni redox activation and catalytic turnover, additional control experiments were performed to directly verify oxygen evolution under the operating conditions used for PEC studies and mapping, as discussed below and detailed in Section 2 in the SI.

### Evaluation of the PEC mapping resolution

**Fig. 2:**
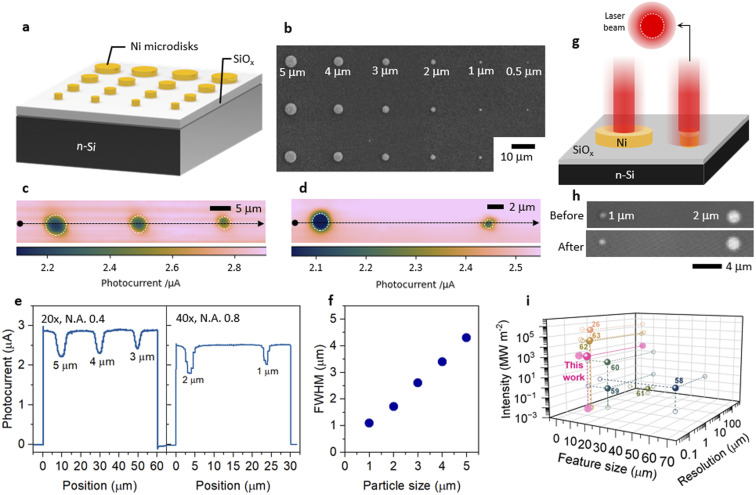
(a) Scheme and (b) SEM top-view image of a n-Si/SiO_*x*_/Ni-mds photoanode. (c and d) Photocurrent maps and corresponding (e) photocurrent profiles of (c) 5 µm, 4 µm, 3 µm Ni-mds (20× objective lens) and (d) 2 µm and 1 µm Ni-mds (40× objective lens). (f) Full width at half maximum (FWHM) value of the photocurrent pit at Ni microdisks with different particle diameters. Applied potential: 1.5 V, step size: 500 nm, laser intensity: 5% AOTF. (g) Scheme of the laser beam illuminating a large and a small Ni-md particle. (h) Confocal images taken before and after the measurement. N.A. is the numerical aperture value of the objective lens. (i) Comparison chart, relating to the reported order of magnitude of the feature size, resolution (reported or estimated from the numerical aperture of the objective lens and wavelength employed in the studies), and light power density, from previous studies (reference numbers are indicated for each data point) summarized in Table S3.^26,58–63^

It is important to experimentally determine the minimum feature size that can be analyzed under our conditions. Here, we present the tests performed on a n-Si/SiO_*x*_ photoanode coated by Ni microdisks (Ni-mds, with Ni thickness of 40 nm) spaced by 20 µm and having a diameter ranging from 5 to 0.5 µm (see Fig. 2a and the Methods section in the SI for more details), these photoanodes are denoted as n-Si/SiO_*x*_/Ni-mds. In this section, the measured photocurrent and associated reactivity will not be discussed; we only employed the measured PEC maps to investigate the resolution of the PEC mapping system. A detailed explanation of PEC maps will be given in the succeeding sections. Fig. 2b shows a SEM top-view picture of the Ni-mds. In this study, low %AOTF was utilized, typically not more than 5%, to avoid potential damage to Ni-mds caused by the intense local illumination. In Fig. 2c, a photocurrent map comprising 5, 4, and 3 µm Ni-mds obtained using a 20× objective lens and a 500 nm step size (*i.e.*, the distance between two illumination points) is shown and presents a good resolution. It is worth mentioning that the photocurrent measured with the local light illumination on Si/SiO_*x*_ surface was higher than on Ni layer. More information and explanation to this result will be given in the following sections. Confocal images were taken before and after the mapping to ensure that the particles were not damaged during the procedure, as shown in Fig. S6. The photocurrent profile reveals that the change of photocurrent intensity decreases as the particle becomes smaller (Fig. 2e and S7) and that the FWHM of the photocurrent pit becomes larger than its actual size with a decreasing microdisk size, as shown in Fig. 2f. This is due to the beam diameter (theoretical size of 800 nm obtained with 20× objective lens, N.A. 0.4, wavelength 633 nm, see Methods section in the SI for the calculation) that becomes comparable to the Ni-md size. In fact, the actual illumination area is expected to be greater than the theoretical diameter of the laser because the intensity distribution of the beam used in this work is Gaussian.^55–57^ Thus, when the laser is located on the Ni-md, part of the light is also illuminating the Si surface, as illustrated in Fig. 2g. Under these conditions, the smallest Ni-md that can be satisfactorily mapped is 3 µm, and attempts to image smaller Ni-mds were not successful.

To reach a smaller Ni-md size, the effects of the objective lens and the step size were studied. As can be seen in Fig. 2d, changing the objective lens to 40× (0.8 N.A.) allowed the acquisition of the PEC mapping with 2 µm and 1 µm Ni-mds reliably without any observable damages (Fig. 2h). Corresponding photocurrent maps without interpolation or smoothing effects can be found in Fig. S8. Decreasing the step size to 200 nm increased the resolution of the raw photocurrent map (Fig. S8c). However, the fluctuation in the photocurrent during the measurement was much higher since the measurement took a longer time compared to larger scanning step sizes. To sum up, these results show that our PEC mapping setup can reliably differentiate PEC response by scanning structures down to the µm size. By comparing our procedure to previous PEC mapping based on SPCM reports on Si-based and other photoelectrodes (summarized in Tables S2, S3 and Fig. 2i), we can state that we have achieved a comparable resolved photocurrent map on our Si photoanodes. The key distinction of our setup is the strong light intensities, allowing the study of phenomena that were previously not accessible. In the following, larger features are investigated with the 20× objective lens and a step size of 1–2 µm.

### Effects of the coating and the electrolyte immersion

**Fig. 3:**
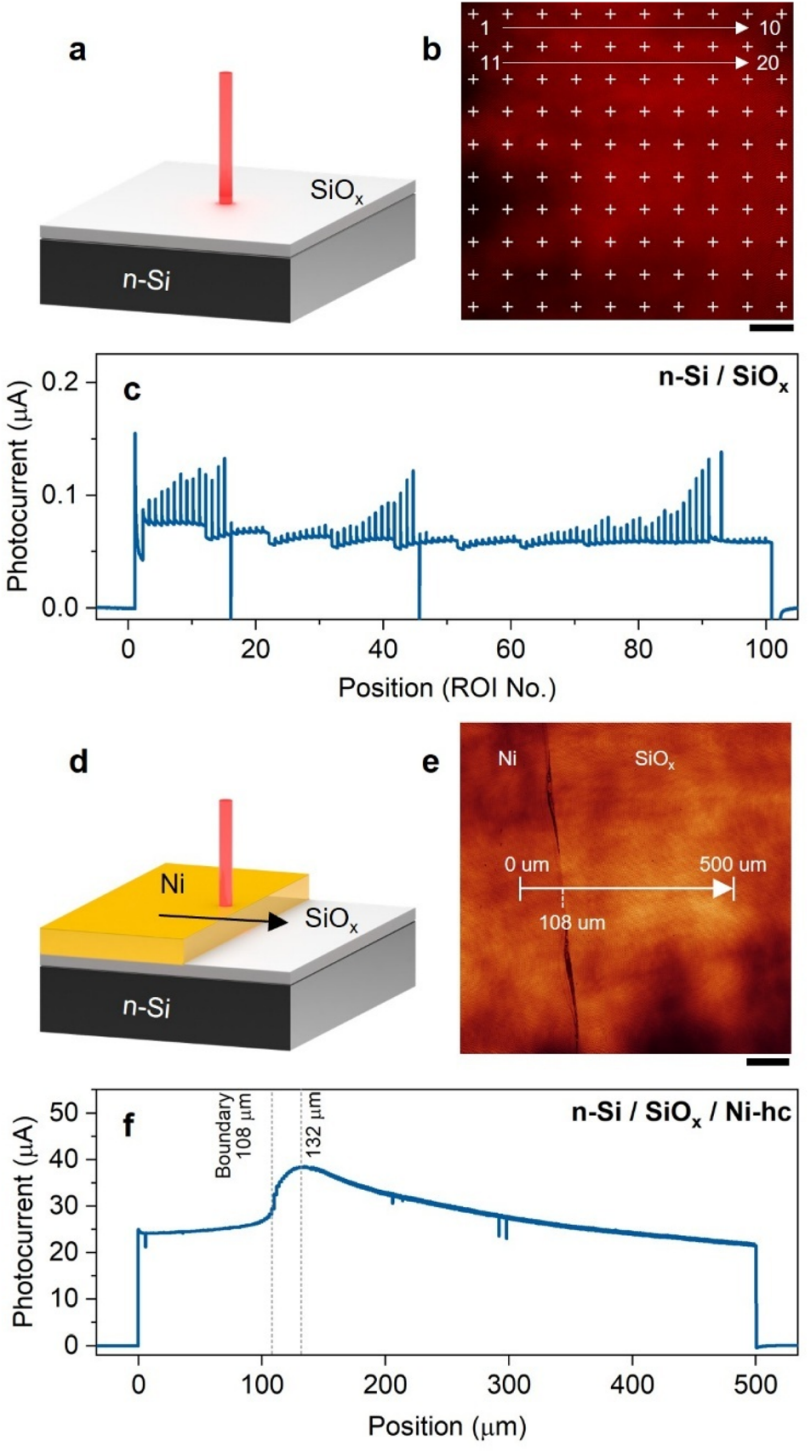
(a and d) Schemes of the photocurrent measurements on (a) n-Si/SiO_*x*_ and (d) n-Si/SiO_*x*_/Ni-hc. (b and e) Confocal images and regions of interest (ROIs, cross mark or line) (equivalent to illumination points) employed on (b) n-Si/SiO_*x*_ and (e) n-Si/SiO_*x*_/Ni-hc. (c and f) Photocurrent profiles measured on (c) n-Si/SiO_*x*_ and (f) n-Si/SiO_*x*_/Ni-hc. Applied potential: 1.5 V, step size: 2 µm, laser intensity: 20% AOTF. The scale bars equal 100 µm.

It has often been shown in the literature that Si-based MIS photoanodes with inhomogeneous junctions exhibit better PEC performance compared to their homogeneous counterparts.^64^ This raises the question of how different regions of the photoanode (metal-coated or uncoated areas) contribute to the PEC performance. In this section, we study photocurrent profiles of a series of photoanodes with different macroscale coatings at 1.5 V under local illumination with 20% AOTF. First, we note that the overall photocurrent recorded at an uncoated n-Si/SiO_*x*_ photoanode, shown in Fig. 3a–c, is significantly lower (<0.1 µA) compared to that of a photoanode modified with a conformal Ni full coating (thickness of 40 nm), denoted here as n-Si/SiO_*x*_/Ni-fc (Fig. S5d) or n-Si/SiO_*x*_/Ni-mds (Fig. 2e and f), even when a lower illumination intensity was used (Fig. S5e–g). This is expected since the surface of n-Si/SiO_*x*_ is known to be a poor conductor and a bad OER catalyst.^65^ Then, a photoanode half-covered with a Ni layer (Ni thickness = 40 nm), denoted n-Si/SiO_*x*_/Ni-hc, was investigated by illuminating different areas, *i.e.*, by moving the light spot starting from the Ni-covered side to the bare SiO_*x*_ surface as shown in Fig. 3d and e. It is worth noting that the photocurrent measured originates from the whole surface of the photoanode and relates to the faradaic process, mainly OER in our case.

Comparing the results obtained in Fig. 3c (*i.e.*, negligible photocurrent produced on n-Si/SiO_*x*_) with that of Fig. 3f, it becomes clear that the photogenerated h^+^ are not confined to the illuminated area only and that minority carriers photogenerated in n-Si/SiO_*x*_ can be collected over several hundred µm from the illumination site before being consumed for the electrochemical reaction occurring at the Ni/electrolyte interface. This is first corroborated by the charge transport properties of single-crystalline Si, which allows for a very high carrier diffusion length to be achieved. Indeed, as documented in Section 3 of the SI, we calculated a diffusion length *L*_p_ of 350 µm for h^+^ generated in the bulk, according to the doping level of the Si wafer used in this work. While high photocurrent values were measured on both areas (Ni and SiO_*x*_) Fig. 3f exhibits strong differences between these two regions. Namely, the photocurrent is quasi-stable (25 µA) for the Ni-coated region and increases (up to 38 µA) as the laser transitions from the Ni edge to the bare SiO_*x*_ surface. The highest photocurrent value was obtained around the boundary between these two regions and started to drop as the laser was displaced further away from it. The lower activity of the Ni region compared to the SiO_*x*_ region is attributed to the transmission loss induced by the 40 nm Ni thin film (see the transmittance spectrum in Fig. S9). We note that this is in good agreement with what was measured for the n-Si/SiO_*x*_/Ni-mds photoanodes (Fig. 2) which also revealed a photocurrent loss upon illumination on the Ni-mds and the fact that thinner Ni films produced higher photocurrents (Fig. S10 and Table S4). A remarkable feature of Fig. 3f is the photocurrent decay along the distance on SiO_*x*_, which indicates, in these conditions, a decrease of the charge collection probability as the carrier generation site moves away from the OER-active Ni coating.

To evaluate whether photogenerated holes originating from regions not directly in contact with the electrolyte can contribute to electrochemical charge transfer, we performed control experiments in which selected areas of the photoanode were electrically isolated from the electrolyte using a transparent Kapton layer (Section 4 in the SI). These experiments revealed a clear contrast between Ni-coated and bare Si/SiO_*x*_ regions. When illumination was applied to Ni-covered regions not exposed to the electrolyte, a sustained photocurrent was still observed, indicating efficient long-range hole transport toward catalytically active Ni/electrolyte interfaces over millimeter-scale distances. In contrast, illumination of bare n-Si/SiO_*x*_ regions not in contact with the electrolyte yielded negligible photocurrent, especially in the long-range, demonstrating that photogenerated holes in these areas cannot be effectively collected. These results indicate that long-range lateral charge collection strongly depends on the presence of the Ni layer. With Ni present, long-range transport is enabled either through a permanent MIS junction facilitating subsurface hole transport or *via* transport through the conductive Ni layer itself. In the absence of Ni, lateral hole collection relies on the space-charge region formed only when n-Si/SiO_*x*_ is directly interfaced with the electrolyte. Similar inversion-layer–mediated lateral transport has previously been reported in Si-based photoelectrodes, supporting this interpretation.^44^

In our system, we have shown that illumination on bare n-Si/SiO_*x*_ does not result in effective hole transfer to the electrolyte (Fig. 3a), demonstrating that the illumination site itself is not electrochemically active. Combined with the observation that long-range hole collection requires the presence of a space-charge region, it suggests that photogenerated holes do not diffuse conventionally to the Ni catalyst sites, as considered in the framework of the Gärtner model (one-dimensional vertical transport under uniform illumination and local availability of charge-transfer reactions at the illumination site).^47,66^ Additional evidence of long-range charge collection on mm scale, far beyond the diffusion length, was also observed (Fig. S11). Importantly, Ni 2p X-ray photoelectron spectroscopy (XPS) spectra acquired at locations a few millimeters away from the Ni boundary showed no detectable Ni signal, excluding long-range Ni dissolution–redeposition as the origin of this behavior. Instead, holes are first accumulated or guided within the space-charge region and subsequently collected laterally toward catalytically active Ni/electrolyte interfaces.

### Effects of power density and position of the light spot relative to the Ni coating

**Fig. 4:**
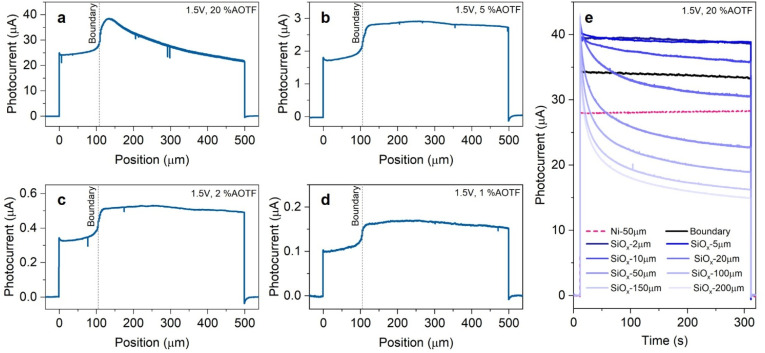
(a–d) Photocurrent profiles measured at n-Si/SiO_*x*_/Ni-hc with a scan from Ni towards SiO_*x*_ using %AOTF of (a) 20, (b) 5, (c) 2, and (d) 1. (e) Photocurrent measured during a long (5 min) local light illumination (at 20% AOTF) on Ni and SiO_*x*_ at different distances from the boundary (the distance from the boundary is indicated in the legend). Applied potential: 1.5 V.

The behavior observed at n-Si/SiO_*x*_/Ni-hc in Fig. 3f is now studied in more detail. To verify the distance dependency, we first performed similar experiments with lower light power densities. In Fig. 4a–d, it is found that the dependency of the photocurrent on the distance from the boundary is only observed when using a high-intensity light spot (20% AOTF). The overall photocurrent is higher when increasing the light power density and, with light intensity < 20% AOTF, the photocurrent is found to be higher on SiO_*x*_ compared to Ni, which is, again, caused by the light transmission loss through the metal thin film. We think that the behavioural change at high intensity is the result of excessive carrier injection in the Ni and band flattening, caused by an excessive density of photogenerated carriers and/or modification of the junction.^67–69^ Since the illumination power density is extremely high (see Table S1), we presume that the evolution of the physical and chemical properties of the Si interface is possible, some studies have shown that the conversion of Si to its oxide species can be induced by high-power laser light.^70–72^

To verify this assumption, we performed long (5 min) local illumination photocurrent measurements at different distances from the boundary with a fixed applied potential (1.5 V). The results, shown in Fig. 4e, indicate that the photocurrent characteristics during a long illumination period also change with the boundary-laser distance. At locations far from the boundary (>10 µm), the photocurrent decay is prominent, and a steady state profile is observed after a few minutes of illumination (see also Fig. S14a), which could be indicative of charge accumulation and/or self-limiting PEC SiO_*x*_ growth, consuming the photogenerated h^+^ mainly during the early stage of illumination. Additionally, CVs recorded under local light illumination with 5% AOTF and 20% AOTF at different areas were recorded, as shown in Fig. S15. This CV study indicates a similar trend to that found with the potentiostatic measurements of Fig. 4a ande, as noticed by the evolution over several scan cycles and the shift in the onset potential. We postulate that this is caused by the junction modification occurring at the SiO_*x*_ interface, *e.g.*, the oxidation to a thicker and denser SiO_*x*_ layer. Indeed, irreversible Si oxidation is supported by the higher photocurrent generation during the first cycles of intermittent illumination at a constant applied potential (Fig. S14a). Also, in other works, a thick HfO_2_ insulator layer was found to influence the junction properties, *i.e.*, lowering the barrier height and thus reducing the thickness of the space charge layer.^73^ This leads to a higher probability of charge recombination, fewer h^+^ being transported to Ni surface, and, *in fine*, a decrease in photocurrent. The additional formation of SiO_*x*_ under Ni is not expected because a thickness of 40 nm should shield the Si surface from the electrolyte. Despite observing a clear change in the PEC properties, we were not able to detect by SEM an obvious morphological change related to light-induced SiO_*x*_ formation on the illuminated Si area (see Fig. S16). Indeed, this change is expected to be subtle and difficult to probe by conventional surface characterization methods. Although it has been reported that laser illumination can be used to structure Si,^71^ our characterizations suggest that no observable physical damages (at the scale of study) were made on the Si surface under the conditions of our experiments.

Even if the local light intensity is high for 20% AOTF, and that, according to Beer–Lambert law (Fig. S17), a significant part of the photon density is expected to reach beyond the depletion width (on the order of 1 µm, see Section 3 in the SI), photogenerated minority carriers are still expected to be collected efficiently due to the long diffusion length (*L*_p_ = 350 µm), provided that the junction properties remain unchanged and the charge-transfer reaction is not limited. To assess whether the observed photocurrent behavior could be attributed to this diffusion-limited collection regime, we analyzed the photocurrent–light intensity relationship. Under Gärtner behavior, the photocurrent should scale linearly with light intensity as long as carrier collection remains efficient. We first performed this analysis on the Ni-covered regions, which serve as a reliable baseline due to the presence of a stable MIS junction. As shown in Fig. S18a, a linear photocurrent–intensity relationship is preserved over the entire intensity range employed in this study. This result indicates that, in our experimental conditions, the total photogenerated carrier density does not exceed the diffusion-limited collection capacity of the junction, when only Ni is illuminated. We then analyzed the photocurrent–intensity relationship when illuminating the Ni-free regions. When early-time photocurrent values (∼1 s) are considered under high-intensity illumination (20% AOTF) and a relatively fresh interface, the photocurrent exhibits an approximately linear dependence on light intensity, with only minor deviations as the illumination distance increases, as shown in Fig. S18b. This indicates that, at short times, photogenerated holes can still be efficiently mediated by the space-charge region and collected at the Ni catalyst. In contrast, in Fig. S18c, after prolonged high-intensity illumination under applied bias (5 min at 1.5 V), the modified surface exhibits a pronounced deviation from linearity at high light intensity, which becomes more severe with increasing distance from the Ni boundary. This behavior indicates that irreversible modification of the Si/SiO_*x*_ interface permanently degrades the efficiency of two-dimensional lateral charge collection and amplifies the distance-dependent transport limitations observed in the system.

Due to the use of a buffered electrolyte, we do not consider the influence of local pH gradients that may arise due to OER, however, the high density of carrier injection toward the Ni catalyst sites is expected to further increase recombination losses. As previously mentioned, in addition to SiO_*x*_ growth, under strong local illumination, high accumulation of photogenerated carriers (suggested by the repetitive transient decay in Fig. S14a) and ions at the semiconductor–electrolyte interface can reduce interfacial band bending, leading to less efficient charge separation and enhanced recombination. Consequently, although holes photogenerated deep within n-Si can be, in principle, efficiently collected under the illumination intensities employed (due to the high *L*_p_ value) their contribution to OER decreases at large distances from the Ni boundary when junction-mediated lateral charge collection becomes inefficient due to charge accumulation and interfacial modification.

Owing to the evolution of the photoanode during operation, the individual contributions of concurrent processes cannot yet be fully decoupled. While the present experiments clearly suggests that irreversible modification of the Si/SiO_*x*_ interface gives rise to the distance-dependency behavior under high-intensity illumination, the role of reversible charge accumulation and carrier injection remains less conclusive. Future studies employing more chemically stable dielectric layers or alternative MIS architectures could help decouple the contributions of reversible charge accumulation and permanent interfacial evolution to lateral transport behavior.

### Photogenerated holes transport and PEC mapping of the Ni/SiO_*x*_ boundary

**Fig. 5:**
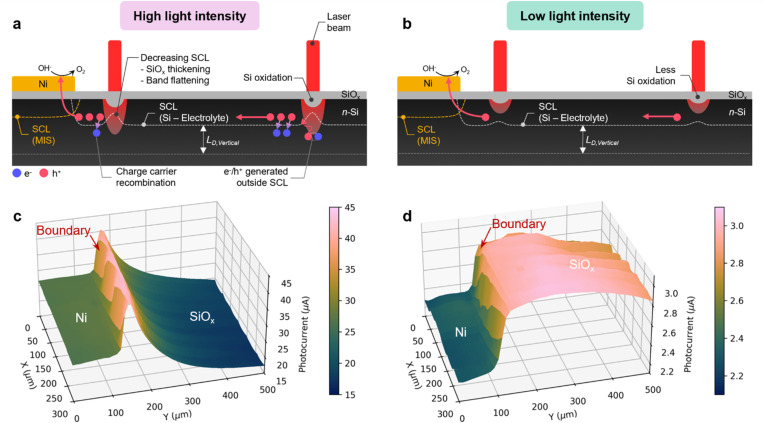
(a and b) Schemes displaying the possible photogenerated charge diffusion/transfer pathways under local illumination with (a) high and (b) low light intensity. (c and d) 3D Photocurrent maps obtained under laser intensities of (c) 20% AOTF and (d) 5% AOTF. Step size: 2 µm during the mapping. Applied potential: 1.5 V. 2D Photocurrent maps overlaid with the confocal image can be found in Fig. S19. SCL: space charge layer, *L*_D,Vertical_: vertical minority diffusion length. The determination of the relative space charge/depletion width between bare Si and Ni-covered region is discussed in Fig. S20.

The key charge transport processes determined from the results of the previous sections are illustrated in Fig. 5a and b for high and low power density illumination. At high light intensity, Fig. 5a indicates several pathways affecting the photocurrent changes with the position of the laser spot relative to the Ni boundary. The photocurrent-boundary distance dependency is the result of several phenomena (discussed in the previous section) that decrease the h^+^ lifetime at high illumination intensity. Thus, an h^+^ generated far from the boundary has less chance of reaching the Ni area. In the case of weaker light intensity (Fig. 5b), the photogenerated holes are efficiently transferred through the space charge layer, this is evident from the independence of the collected charge regardless of the illumination position.

The photocurrent maps, recorded for two illumination power densities (20 and 5% AOTF) are shown in Fig. 5c and d. As shown earlier in Fig. 3f, for the high-power regime, the photocurrent considerably increases when the light spot is located on SiO_*x*_ near the boundary and then decreases with the distance. The map of Fig. 5c shows that this behavior occurs similarly over the whole investigated area. For the lower illumination power, Fig. 5b shows that the photocurrent is higher over the whole SiO_*x*_ area and does not depend on the distance from the boundary. Though, even at a long range, the overall photocurrent under high intensity illumination is still higher compared to lower intensity. It is worth noting that these maps were recorded with a relatively short illumination time of 1 s, thus, with the high laser intensity, the photocurrent is far from the steady state. According to Fig. 4e, at such a high illumination power density, for a longer illumination, an even greater current step is expected between the two regions. To sum up, in the two last sections, we have rationalized the PEC maps obtained at photoanodes covered by macroscale Ni coatings (n-Si/SiO_*x*_/Ni-hc). Next, we will investigate the behavior at micrometric Ni patches.

### PEC mapping of patterned MIS photoanodes

**Fig. 6:**
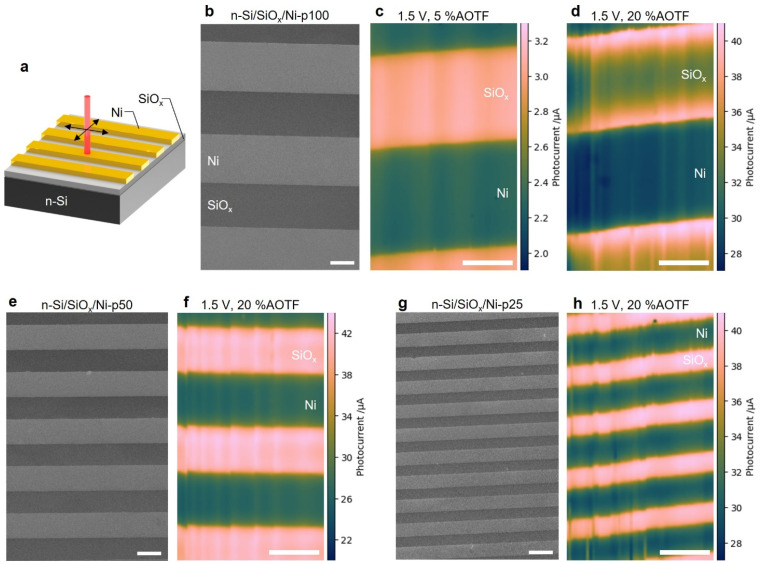
(a) Scheme displaying the structure of n-Si/SiO_*x*_/Ni-p*Θ*, where *Θ* is the width of the microband in µm. SEM images of (b) n-Si/SiO_*x*_/Ni-p100, (e) n-Si/SiO_*x*_/Ni-p50, and (g) n-Si/SiO_*x*_/Ni-p25. Photocurrent maps of n-Si/SiO_*x*_/Ni-p100 obtained with (c) 5% AOTF and (d) 20% AOTF; (f) n-Si/SiO_*x*_/Ni-p50 with 20% AOTF; and (h) n-Si/SiO_*x*_/Ni-p25 with 20% AOTF. Step size: 2 µm. Applied potential: 1.5 V. The scale bars equal 50 µm.

Another series of electrodes was produced by patterning them with 40 nm-thick Ni microbands (Fig. 6a and see Methods section in the SI for more details), these MIS photoanodes are referred to as: n-Si/SiO_*x*_/Ni-p*Θ*, with *Θ* designating the width of the microband and the pitch between microbands, in µm units. Top-view SEM images of n-Si/SiO_*x*_/Ni-p100, n-Si/SiO_*x*_/Ni-p50 and n-Si/SiO_*x*_/Ni-p25 are shown in Fig. 6b, e, and g, respectively. These photoanodes were also tested for “conventional” OER, that is, under simulated sunlight (AM 1.5G, 100 mW cm^−2^). The corresponding CVs, shown in Fig. S22, revealed that they all promoted efficient OER.

We first recorded PEC maps at n-Si/SiO_*x*_/Ni-p100 with an applied potential of 1.5 V. Fig. 6c shows that, with a low illumination power density (5% AOTF), the map exhibits a photocurrent which is higher on SiO_*x*_ and stable in both regions (SiO_*x*_ and Ni). Conversely, at high illumination power density (20% AOTF), Fig. 6d reveals that the photocurrent is much stronger at the boundary between SiO_*x*_ and Ni. The photocurrent maps obtained from these patterns highlight that, in the case of a (conventional) full photoelectrode illumination, the uncoated SiO_*x*_ surface exhibits a higher contribution to the overall photocurrent. We note that the photocurrent values are consistent with those previously determined in Fig. 5 on macroscale Ni coatings. The photocurrent map obtained with a lower applied potential of 1.0 V at 20% AOTF also exhibits similar characteristics, as can be seen in Fig. S22. After the photocurrent measurement, we noticed a color change of the entire Ni stripe even though the local illumination only took place in the middle of the stripe, as shown in Fig. S23. We attribute this behavior to the oxidation of Ni which confirms that the whole Ni microband was electroactive during mapping.

The photocurrent maps obtained on photoanodes with narrower and closer microbands (n-Si/SiO_*x*_/Ni-p50 and n-Si/SiO_*x*_/Ni-p25) at 20% AOTF and 1.5 V are shown in Fig. 6f and h. Even if the illumination power density is very high in this case, these maps do not present the characteristic photocurrent overshoot at the boundary between SiO_*x*_ and Ni. We surmise that, in these conditions, because the distance between the Ni patches is considerably lower than the diffusion length of h^+^ (∼350 µm, see Section 3 in the SI), they can collect most of them to efficiently perform OER. Additionally, at this length scale, the deleterious effects of excessive injection, charge accumulation and irreversible modification of the Si/SiO_*x*_/electrolyte junction are minimized, preserving uniform charge collection behavior. All these maps confirm our previous hypotheses and point out the importance of the inter-Ni patch distance at high illumination power density.

## Conclusions

In this work, by using a PEC mapping system with a confocal microscope as the light source, we investigated the photocurrent response of various MIS photoanodes composed of n-Si/SiO_*x*_ coated with Ni catalyst patches of different geometries during OER operation under high-intensity local illumination. We have shown that our mapping method can image structures down to 1 µm and the information provided by this approach is complementary to, yet distinct from, previous reports on similar types of Si photoanodes utilizing SECM, photoconductive atomic force microscopy, and PECL techniques.^18,28,29^ Indeed, our PEC mapping technique allows for resolving the PEC-active regions of the photoelectrode rather than the reaction products or the location of the interfacial charge transfer. We found that high photocurrents were produced with a local illumination of uncoated n-Si/SiO_*x*_ when a Ni patch was present on the surface, suggesting an efficient hole transport from n-Si to Ni surface over a large (mm) distance. We also observed that hole transport under the bare SiO_*x*_ surface relies on the presence of the electrolyte at the interface to create the space charge region that separates charges and mediates the long-range lateral charge collection. On non-homogenously coated photoanodes, where the Ni surface serves as a reaction site, the uncoated n-Si/SiO_*x*_ regions contribute to the saturation photocurrent density to a greater extent by absorbing a larger photon density. An important conclusion of our study is that, under high light intensities, hole transport from the uncoated SiO_*x*_ to the Ni patch becomes less efficient, causing the local photocurrent to decrease as a function of distance to the Ni patch. This limitation, not observed at lower light intensities, reflects a breakdown of efficient lateral hole collection under high-intensity illumination caused by charge accumulation, interfacial modification and excessive charge injection, which reduce the effectiveness of the space-charge-mediated transport and increase recombination losses. Although the individual contributions of these events cannot yet be fully separated, the observed distance dependence emerges only after prolonged high-intensity operation, indicating a key role of junction evolution. Experiments performed with microband arrays demonstrates that this limitation can be compensated by a shorter distance between Ni pads. Compared to previous laser-based SPCM studies, our approach uniquely combines high spatial resolution with high intensity light operation, enabling direct interrogation of non-linear and dynamic charge transport in MIS photoanodes under PEC conditions (Fig. 2i). These findings provide an essential guideline for the design of more efficient PEC-driven solar fuels production systems, operating under extreme light intensities, such as concentrated sunlight.

## Author contributions

K. K.: conceptualization, data curation, formal analysis, investigation, methodology, validation, visualization and writing – original draft. B. G., P. G., V. L., L. S., M. T.: methodology and investigation. P. P.: validation, supervision, funding acquisition, project administration, and writing – review & editing. G. L.: conceptualization, validation, visualization, supervision, resources, funding acquisition, project administration, and writing – review & editing. All authors have approved the final version of the manuscript.

## Conflicts of interest

There are no conflicts to declare.

## Supplementary Material

SC-017-D5SC08974C-s001

## Data Availability

The supporting data is provided in supplementary information (SI). Supplementary information: methods, analysis of the PEC response of a homogeneously-coated photoanode, calculation of minority carriers' diffusion length and depletion width in n-type Si, effect of surface shielding and electrolyte immersion, supplementary tables and supplementary figures. See DOI: https://doi.org/10.1039/d5sc08974c.
