# Supplementary material for: Imaging hole transport at catalyst-coated MIS photoanodes for water splitting under high-intensity illumination
Source: Chem Sci. 2026 Feb 20;17(15):7717–28. doi: 10.1039/d5sc08974c (PMC12935071; doi:10.1039/d5sc08974c)
Supplement: SC-017-D5SC08974C-s001 [file SC-017-D5SC08974C-s001.pdf]

Supplementary Information (SI) for Chemical Science.
This journal is © The Royal Society of Chemistry 2026

Supplementary information

Imaging hole transport at catalyst-coated MIS photoanodes for water splitting under high-intensity illumination

Kanokwan Klahan,[a,b] Bertrand Goudeau,[a] Patrick Garrigue,[a] Véronique Lapeyre,[a] Lionel Santinacci,[c] Mana Toma,[d] Pichaya Pattanasattayavong,[b]* Gabriel Loget[a]*

[a] University of Bordeaux, Bordeaux INP, ISM, UMR CNRS 5255, Pessac 33607, France

[b] Department of Materials Science and Engineering, School of Molecular Science and Engineering, Vidyasirimedhi Institute of Science and Technology (VISTEC), Rayong 21210, Thailand

[c] Aix-Marseille Univ, CNRS, CINaM, Marseille, France

[d] Department of Electrical and Electronic Engineering, School of Engineering, Institute of Science Tokyo, Nagatsuta-cho 4259, Midori-ku, Yokohama, 226-8501, Japan

*Corresponding authors: pichaya.p@vistec.ac.th; gabriel.loget@cnrs.fr

Table of contents

Section 1 - Methods	S2
Section 2 - Analysis of the PEC response of a homogeneously-coated photoanode	S4
Section 3 - Calculation of minority carriers diffusion length (L_p) and depletion width (W) in *n*-type Si	S6
Section 4 - Effect of surface shielding and electrolyte immersion	S6
Section 5 - Supplementary tables	S8
Section 6 - Supplementary figures	S10
Section 7 - References	S22

Section 1 - Methods

Materials and reagents

All silicon (Si) wafers (280 μm-thick) were purchased from University Wafers and were single side polished (*n*-Si, <100>, phosphorus-doped, 1-10 Ω cm resistivity). Boric acid (H_3BO_3, ≥99.5%), sulfuric acid (H_2SO_4, 98%), and hydrogen peroxide solution [H_2O_2, 30%(w/w) in H_2O] were purchased from Sigma-Aldrich. Potassium hydroxide (KOH, AR grade) was purchased from UNIVAR. Acetone (AR grade) and isopropanol (AR grade) were purchased from Carlo Erba.

Sample preparation and characterization

Substrate cleaning and Ni film sputtering

Si surfaces were cleaned by sonication in glass cleaning detergent, deionized water, acetone, and ethanol, each for 10 min in that order. The surfaces were then oxidized in piranha solution (3:1 v/v concentrated H_2SO_4:30% H_2O_2) at 105 °C for 30 min, leading to *n*-Si/SiO_x. Ni thin films were deposited on the *n*-Si/SiO_x substrate by sputtering with a Leica EM ACE600 coating system (the thickness was monitored *in situ* by a quartz crystal microbalance). During sputtering, the Ar gas pressure was 2×10^{-2} mbar and the current was 100 mA.

Preparation and characterization of patterned Ni thin films

The patterned Ni film was produced on the *n*-Si/SiO_x surface by photolithography followed by lift-off. First, a positive photoresist film (Microposit S1813) was deposited onto *n*-Si/SiO_x via spin coating and heated at 115 °C for 1 min. Using a UV-Kub 2 (Kloé) masking system, the surface was exposed to UV light through a photolithographic mask. After exposure, the sample was developed in Microposit MF-319 and rinsed with water. Ni film was then sputtered onto the patterned photoresist film. By sonicating in acetone, the photoresist film was removed, producing the desired patterned Ni thin film on the *n*-Si/SiO_x substrate. The Ni microdisks array was produced by e-beam lithography, as described in our previous report.[1]

Physical and surface characterizations

The morphology of the Si photoanodes was studied by scanning electron microscopy (SEM) using a TESCAN VEGA3 SBH instrument. For atomic force microscopy (AFM) measurements, a Bruker MultiMode 8 (ScanAsyst air method) was employed to determine the thickness of Ni film (**Figure S4**). The AFM probe was a Bruker model SCANASYST-AIR (70 kHz, 0.4 N m^{-1}, 115 μm length). The transmittance spectra of the Ni film and Kapton tape were measured using an Agilent Cary 100 UV-visible spectrophotometer.

Electrode preparation

A large Ohmic contact was formed at the backside of the photoelectrode by scratching the surface, applying InGa and silver paint, and then covering the whole surface with Cu tape. Thus, the collection of electrons is not expected to be a limiting factor during the experiments.

Photoelectrochemical (PEC) mapping system

An inverted confocal microscope (Leica DMi6000B CS TCS SP5) was used as the primary component of the PEC mapping system, providing a focused laser source for both imaging and photogeneration. The electrochemical cell was an a-Clipse P (Idylle), which featured a bottom glass window and an electrolyte channel (thickness of ~3 mm). The geometrical electrochemical surface area was defined by the O-ring diameter and equaled 0.41 cm^2. In the case of photoelectrodes patterned with Ni microbands, about 50% of the patterned area was covered by the Ni thin film. The pattern size was a square of 0.5×0.5 cm^2. The built-in FRAP wizard of the LAS AF program was utilized to control the position of the laser, and the mapping points/grid on the photoelectrode were defined *via* the program interface. The illumination time for each point for PEC mapping was 1 s. The objective lens and the laser used in this study were, respectively, 20× with a numerical aperture (N.A.) of 0.4 and 633 nm from HeNe lasers (unless otherwise specified), giving a laser beam with a theoretical diameter of ~800 nm according to Abbe's equation ($D = \lambda/2N.A.$); however, the real size of the laser beam is expected to be slightly larger, as discussed in the **Results and Discussion Section**. Before PEC measurements, the stage was moved in the XY direction (planar) to select the mapping area while monitoring the optical image of the surface. To ensure an optimal focus of the light spot onto the photoanode, the objective lens was moved in the Z direction (up and down) while the photocurrent (i_{ph}) was recorded using a Palmsens4 potentiostat [with an acousto-optic tunable filter level (%AOTF) of 100]. The working distance was set for the Z coordinate by recording the brightness of the images and the recorded current. The image size and desired resolution were controlled by defining the number of illumination points and the scanning step size. It is worth noting that the illumination area was the same as the laser beam size and not related to the scanning step size. Photocurrent measurements were performed under potentiostatic control. The photocurrent measured from each illumination point was treated as a single pixel when converting the data to a map. Various laser powers were used in this work by adjusting the %AOTF value. The laser power was measured using a Gentec-EO Maestro power meter as a function of %AOTF, and the intensity obtained with the 5× objective lens is shown in **Table S1**. The electrolyte used in this study was 2 M potassium borate buffer (KBi, pH 9.6) unless otherwise specified. The reference and counter electrodes were Hg/HgO and a graphite rod, respectively. All potentials in this article are referred vs Hg/HgO, 1 M KOH. The electrolyte flow was controlled using a peristaltic pump (Grothen).

To determine the optimal region of investigation of our setup, the local PEC response of *n*-Si/SiO_x covered by a conformal Ni full coating (thickness of 40 nm), denoted here as *n*-Si/SiO_x/Ni-fc, was studied across the entire XY path of the confocal microscope (see **Figure S5a** for the illumination grid, area size: 500×500 μm^2) at a potential of 1.5 V and with various %AOTF light intensities as shown in **Figure S5d-g**. The deviation of the photocurrent across the surface of the photoanode, which can be observed in **Figure S5b-c**, was thought to originate from the microscope optical defects producing uneven light intensity across the surface, especially on the edges of the image. This information was used to select the appropriate mapping area to minimize the photocurrent deviation, as indicated by a square in **Figure S5c**. Thus, this area was selected for the PEC mapping experiments.

Section 2 - Analysis of the PEC response of a homogeneously-coated photoanode

We studied the behavior of *n*-Si photoanodes covered by a uniform Ni full coating (thickness of 40 nm, ~50% transmission at 633 nm), denoted here as *n*-Si/SiO_x/Ni-fc, under local illumination. **Figure S1a** presents cyclic voltammograms (CVs) that were recorded under a single-spot local illumination on Ni surface at the center of the sample with different light power (from 0.9 µW to 1.1 mW) by varying an acousto-optic tunable filter level, %AOTF (see **Methods Section** in the supplementary information for more details). In the dark, the anodic current is relatively low (<10 µA) and the CV presents a cathodic peak in the backward scan, which starts at 0.8 V. Under illumination, the anodic current increases, demonstrating that photogenerated h^+ participate in the oxidation processes, and the cathodic peak increases. On the contrary, in **Figure S1b**, the CVs measured under identical conditions on a bare *n*-Si/SiO_x indicate poor h^+ utilization and low catalytic activity with negligible current increase under local light illumination. **Figure S1a** shows that the oxidation current strongly depends on illumination power and reveals the presence of a redox peak, occurring between ~0.6 V and ~0.9 V for both 50 and 20 %AOTF. According to the literature, we attribute this peak to the oxidation of the $Ni^{II}(OH)_2$ present on the Ni thin film surface to $Ni^{III}OOH$.[2] The redox process is quasi-reversible, and the $Ni^{III}OOH$ is reduced back to $Ni^{II}(OH)_2$ at the reduction peak during the backward scan. Under illumination, the oxidation peak is followed by an anodic current, revealing that OER is occurring after the $Ni^{II}(OH)_2$ is converted to $Ni^{III}OOH$, in good agreement with what is expected from the literature.[3]

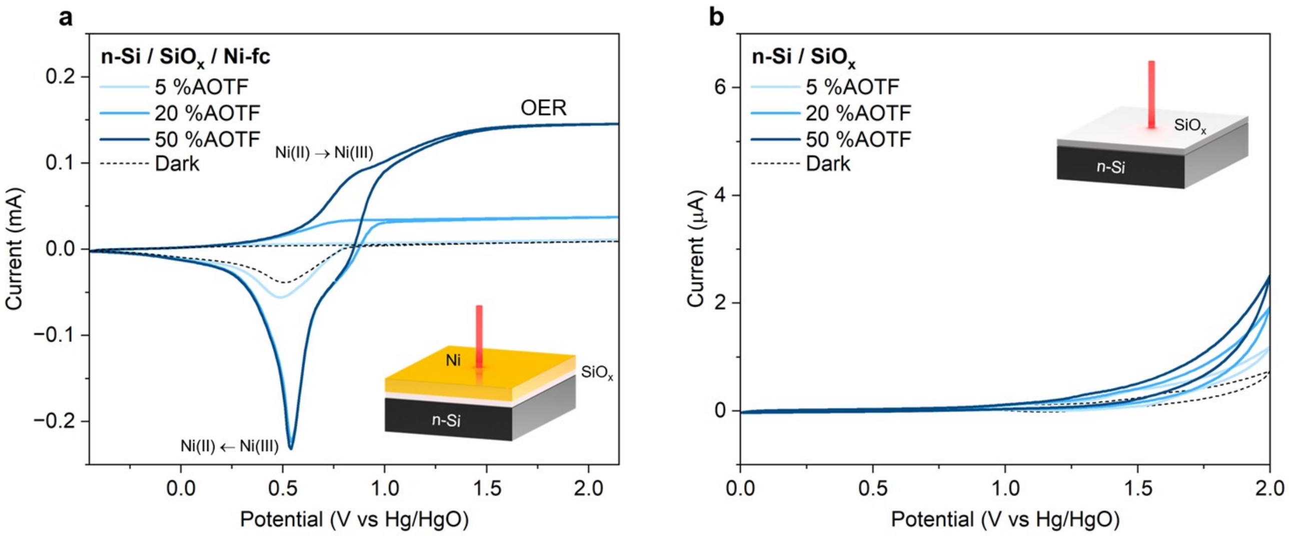

Figure S1. CVs recorded at (a) *n*-Si/SiO_x/Ni-fc and (b) *n*-Si/SiO_x under local illumination at various illumination power densities. Scan rate: 100 mV s^{-1}.

Since most experiments in this work were performed at an applied potential of 1.5 V vs. Hg/HgO, for which oxygen evolution cannot be -*a priori*- assumed in Ni-based systems, additional experiments were conducted to provide direct evidence of gas evolution at the photoanode surface. **Figure S2a** shows the photocurrent recorded while a fully Ni-covered photoanode was biased at 1.5 V under localized illumination during confocal imaging. A stable anodic photocurrent is sustained throughout the measurement. As shown in **Figure S2b**, gas bubble nucleation and growth are directly observed on the

working electrode surface after polarization and continue to evolve with time. Given the anodic bias, electrolyte composition, and cell configuration, hydrogen evolution at the working electrode is excluded; therefore, the observed gas evolution is attributed to oxygen evolution on the Ni surface.

To further distinguish oxygen evolution from Ni redox activation, control oxidation–reduction pulse experiments were performed at 0.9 V, corresponding to the Ni^{2+}/Ni^{3+} redox region. Under all conditions (dark, 5% AOTF, and 20% AOTF), the anodic current exhibits a transient spike that decays within a short time frame (**Figure S2c**), consistent with finite oxidation of electrochemically addressable Ni sites. Illumination accelerates the transient but does not qualitatively alter its finite nature. In contrast, long-term chronoamperometric measurements at 1.5 V under low-intensity illumination (5% AOTF) reveal a stable photocurrent sustained for over 20 minutes (**Figure S2d**), which cannot be accounted to Ni redox activation alone. Taken together, the sustained photocurrent response and the direct observation of gas evolution provide strong evidence that oxygen evolution occurs during PEC operation at this applied potential.

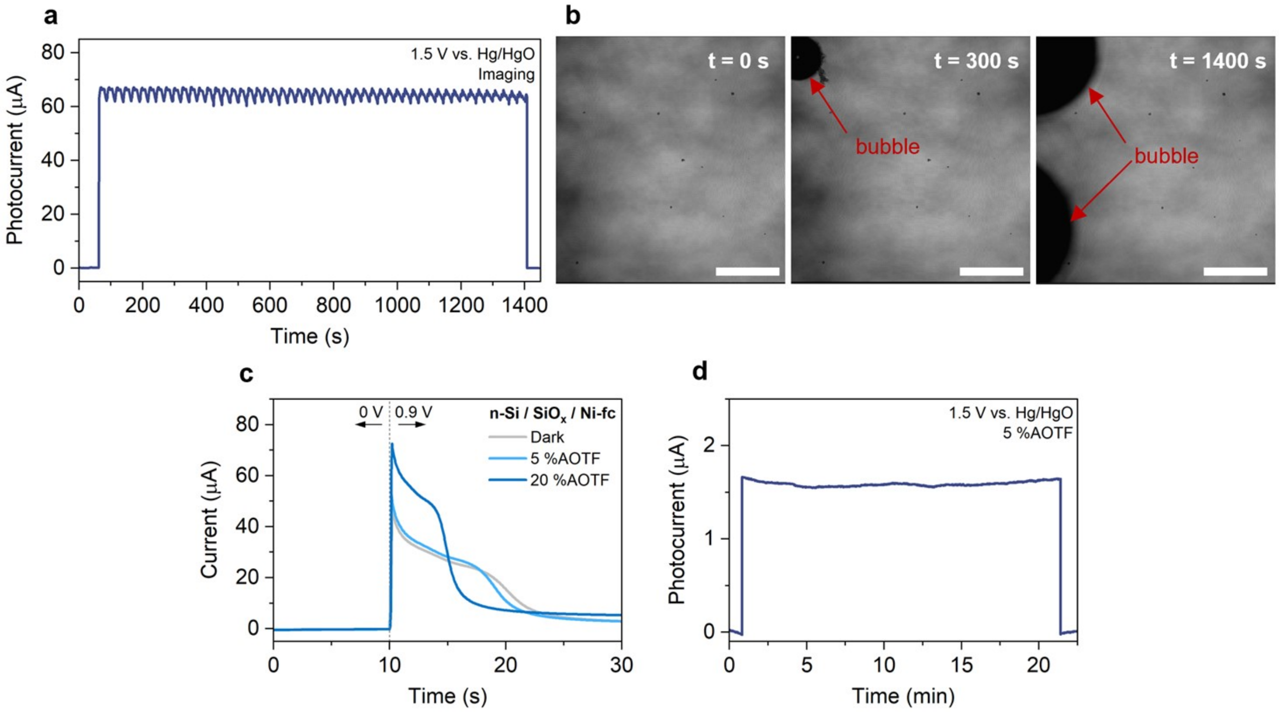

Figure S2. (a) Photocurrent measured on *n*-Si/SiO_x/Ni-fc at 1.5 V during the continuous confocal image scanning. (b) Confocal images recorded at the progressing time, in regard to (a). (c) Current recorded on *n*-Si/SiO_x/Ni-fc with the reduction-oxidation pulse under dark conditions and local light illumination. (d) Photocurrent measured on *n*-Si/SiO_x/Ni-fc at 1.5 V under local light illumination of 5 %AOTF. Scale bar equals 200 μm.

Section 3 - Calculations of minority carriers diffusion length (L_p) and depletion width (W) in *n*-type Si

The hole diffusion length, L_p, can be determined using **Eq. 1**:[4]

$$L_p = \sqrt{D_p \times \tau_p} \tag{1}$$

where D_p is the minority carrier diffusivity and τ_p is the hole lifetime.

As the *n*-Si wafer employed in this study had a resistivity of 1-10 Ω·cm, an average value of 5 Ω·cm was used for the calculations. At room temperature (298 K), the dopant density (N_D) and D_p were determined to be 1×10^{15} cm^{-3} and 12.03 cm^2 s^{-1}.[5] τ_p, at this specific N_D, was estimated to be 10^{-4} s according to the literature.[6,7] Thus, the calculated L_p was ~350 μm.

The depletion width of the Schottky junction, W, is estimated using **Eq. 2**:[8]

$$W = \sqrt{\frac{2\varepsilon_{Si}\varepsilon_0(V_{bi} - V_a)}{qN_D}} \tag{2}$$

Where ε_{Si} is the relative permittivity for Silicon (11.7), ε_0 is the vacuum permittivity (8.854×10^{-12} F m^{-1}), V_{bi} is the built-in potential in the Schottky contact, V_a is the applied potential, q is the electron charge (1.6×10^{-19} C). At zero applied bias and considering a V_{bi} ranging from 0.6 to 1.0 V,[9–11] the depletion width would be around 0.5-1 μm. Nevertheless, the space charge region is larger during the operation with external bias.

Section 4 – Effect of surface shielding and electrolyte immersion

We have investigated whether h$^+$ photogenerated in a region not in contact with the electrolyte can be effectively employed for the electrochemical charge transfer. In **Figure S3a**, the bare SiO_x surface (region without Ni film) of a *n*-Si/SiO_x/Ni-hc photoanode was covered with Kapton tape while most of the Ni surface was left exposed to the electrolyte. Since Kapton tape is relatively transparent at 633 nm (see **Figure S17**)[12], carriers should be photogenerated in *n*-Si when illuminated through it. The photocurrent response indicates that most of the h$^+$ generated on the uncontacted Si/SiO_x surface (not in contact with the electrolyte) cannot travel to the Ni surface in order to drive OER (**Figure S3b**). On the contrary, a significant photocurrent was observed when the uncontacted Ni surface was illuminated, which confirms the transparency of Kapton and suggests an efficient h$^+$ transfer to the Ni/electrolyte interface. To further investigate this effect, we compared the behavior of *n*-Si/SiO_x/Ni-fc and *n*-Si/SiO_x photoanodes half-covered with Kapton tape, as shown in **Figure S3c-f**. Again, h$^+$ photogenerated through the Ni surface not exposed to the electrolyte can be transported to the reaction sites (i.e., the Ni surface exposed to the electrolyte) even though the distance is as far as 3 mm (**Figure S3d**) as shown by the sustained photocurrent. However, this phenomenon does not occur on *n*-Si/SiO_x, as shown in **Figure S3e,f**. We

note that the small photocurrent observed when the uncontacted SiO_x area is illuminated in **Figure S3b** might be due to light scattering in the electrolyte (leading to the presence of light in other unilluminated areas). This is supported by the measurements displayed and explained in **Figure S12**. Still, the diffusion of holes from the uncontacted SiO_x surface to the active Ni area is also plausible according to the large diffusion length of the minority carriers. To sum up, these results suggest a difference in charge carrier transport depending on the presence/absence of the Ni thin film. In the areas without Ni, lateral h^+ collection is mediated via the space-charge region near the Si surface, which only forms when *n*-Si/SiO_x is in contact with the electrolyte.[13,14] In the past, the evidence of the lateral carrier transfer through the inversion layer in *p*-Si photocathodes has also been discovered.[15] With the Ni coating, two possibilities can explain the long-range charge carrier transport: *i*) a permanent Schottky junction is formed between *n*-Si and Ni, facilitating h^+ transport in the space charge layer across the sub-surface; and *ii*) photogenerated h^+ are transferred to and transported through the conductive Ni layer.[10,16]

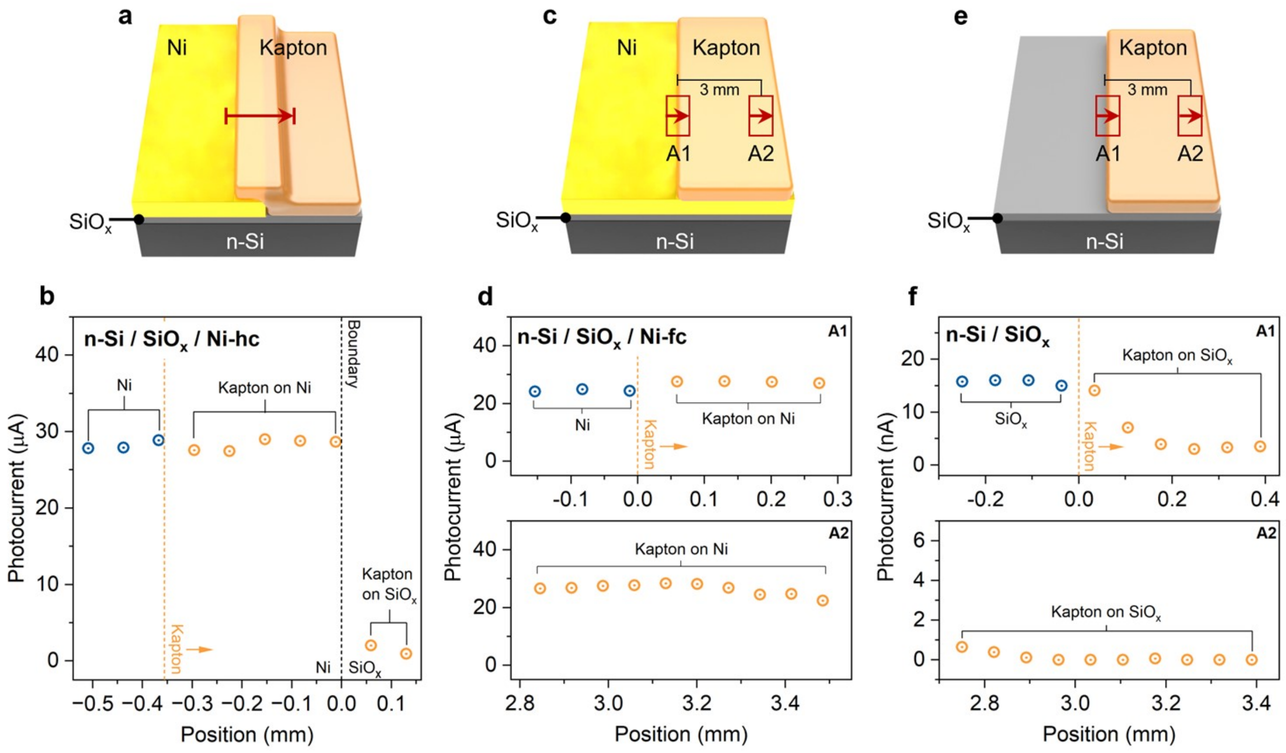

Figure S3. Schemes of the photocurrent measurements and the resulting photocurrent profiles measured on (a, b) n-Si/SiO_x/Ni-hc, (c, d) n-Si/SiO_x/Ni-fc, and (e, f) n-Si/SiO_x, each partially covered with a Kapton tape. Applied potential: 1.5 V, laser intensity: 20 %AOTF. The corresponding confocal images and illuminating points are shown in **Figure S13**.

Section 5 - Supplementary Tables

Table S1. Measured powers and calculated intensities of the 633 nm laser at various %AOTF.

%AOTF	Laser λ = 633 nm		
	Power (μW)	Power density (MW m^{-2})*	No. of suns equivalence
1	0.85	1.7	1.7×10^3
2	2.74	5.6	5.6×10^3
5	14.7	30	3×10^4
20	210	427	4.3×10^5
50	1078	2192	2.2×10^6

*The intensity is calculated with the theoretical laser beam area obtained with 20× lens.

Table S2. Summary table of previous studies related to the photoelectrochemical mapping of Si-based (photo)electrodes.

Surface (Reaction)	Method	Smallest feature*
p-Si/SiO_x/Ti/Pt patches (HER)[15]	SECM** and SPCM (light)	500 μm Pt patch
p^+-Si/Ni microelectrodes (OER)[17]	SECM** (dark)	10 μm Ni microelectrode
n-Si/SiO_x/Ir microbands (ECL)[18]	PECL**	10 μm Ni microband
n-Si/SiO_x/Ni micro-nanoparticles (ECL)[1]	PECL**	50 nm Ni nanoparticle
n-Si/SiO_x/Ni micro-macropattern (OER) – This work	SPCM (light)	1 μm Ni microdisk

*This is referred to as the smallest feature that the authors showed/reported in their studies and might not be the best resolution of their PEC mapping systems.
** These techniques do not measure the photocurrent generated on the surface directly. They instead measure the local product for SECM and local electrochemiluminescence for PECL.

Table S3. Summary table of previous studies employing the scanning photocurrent microscope/mapping on various photoelectrodes.

No.	Sample	Reaction	Setup / Platform	Reported resolution*
1	*p*-WSe_2[19]	HER	Objective lens: 5×, 0.14 N.A. Raman microscope	4 μm beam diameter
2	*p*-WSe_2/WS_2-MoS_2 heterojunction[20]	HER	Objective lens: 100×, 0.55 N.A.	~1 μm beam diameter
3	*p*-WSe_2 monolayer[21]	HER	Raman platform	1 μm beam diameter
4	*n*-TiO_2 nanotubes[22]	OER	Optical fiber (0.22 N.A., 200 μm core diameter)	10 μm feature
5	*n*-TiO_2/Au nanocrystals-nanoparticles[23]	OER	Objective lens: 50×, 0.5 N.A. Optical darkfield microscope	~240 nm Au nanoparticle
6	*p*-Si(micropyramids)/Pt[24]	HER	Objective lens: 50×, 0.5 N.A. Optical darkfield microscope	~2 μm feature
7	*n*-TiO_2/MoS_2/Au nanoplate[25]	OER	Objective lens: 50×, 0.5 N.A. Optical darkfield microscope	~1-10 μm feature
8	*n*-Si/SiO_x/Ni microdisk – This work	OER	Objective lens: 40×, 0.8 N.A. Confocal microscope	1 μm feature

*This is referred to as the smallest feature or beam diameter that the authors showed/reported in their studies and might not be the best resolution of their PEC mapping systems.

Table S4. Photocurrent (i_{ph}) measured at 1.5 V on *n*-Si/SiO_x/Ni-fc with Ni thickness of 20 and 40 nm under local illumination at 20 %AOTF.

Ni thickness (nm)	i_{ph} (μA)
20	28 - 34
40	24 - 30

Section 6 - Supplementary Figures

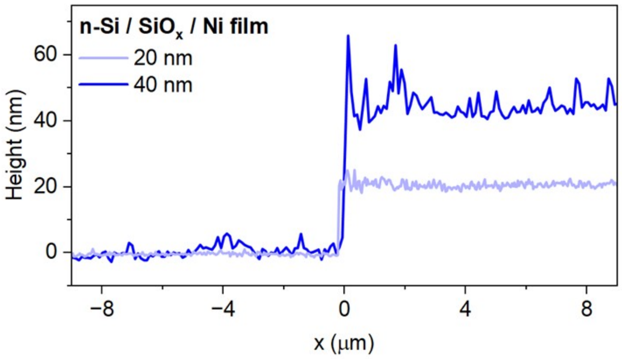

Figure S4. AFM measurements showing the height profile of Ni films deposited on a Si/SiO_x substrate.

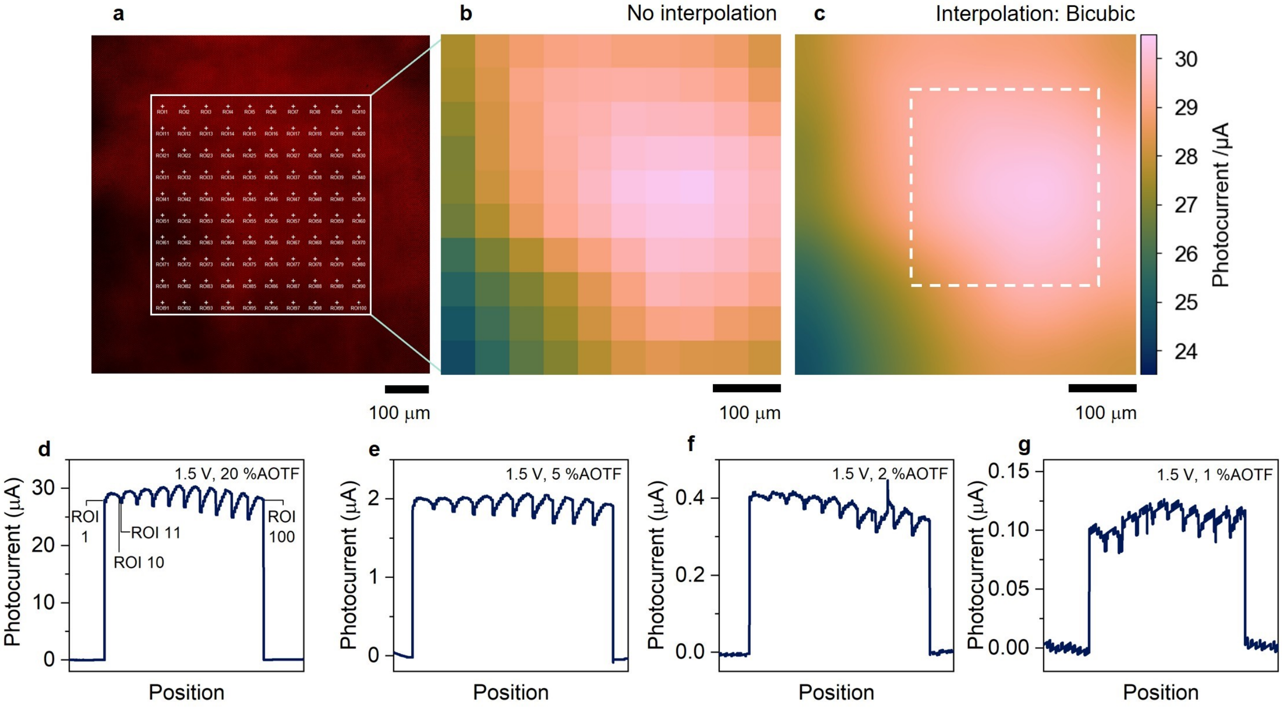

Figure S5. (a) Confocal image indicating the illumination points on a *n*-Si/SiO_x/Ni-fc photoanode. Photocurrent maps corresponding to the illumination points in **Figure S5a** (b) without and (c) with the interpolation/smoothing treatment. The photocurrent was obtained with 20 %AOTF and an applied bias of 1.5 V. (d-g) Photocurrent curves measured at 1.5 V under local light illumination on different points/areas (the illuminating grid shown in **Figure S5a**) with %AOTF of (b) 20, (c) 5, (d) 2, and (e) 1.

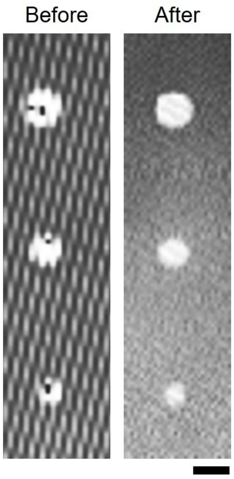

Figure S6. Confocal images of *n*-Si/SiO_x/Ni-mds before and after the mapping measurement. The scale bar equals 5 μm.

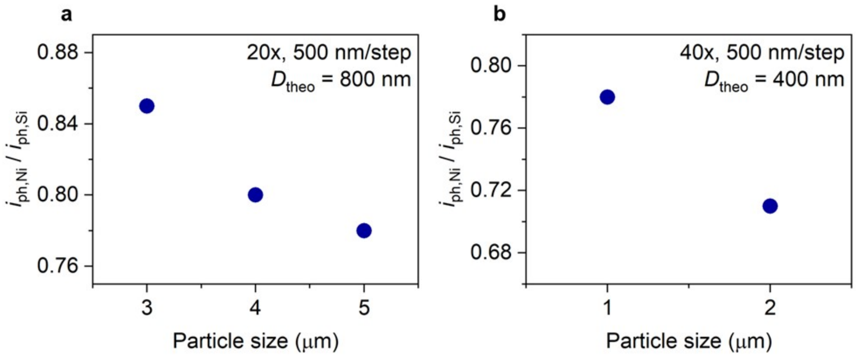

Figure S7. (a)-(b) Ratio of $i_{ph,Ni}$ / $i_{ph,Si}$ measured on *n*-Si/SiO_x/Ni-mds under local illumination on Ni and Si of various Ni particle diameters. $i_{ph,Ni}$ is the lowest photocurrent measured under the illumination on a Ni patch, $i_{ph,Si}$ is the highest photocurrent measured under the illumination on SiO_x, D_{theo} is the theoretical diameter of the laser beam.

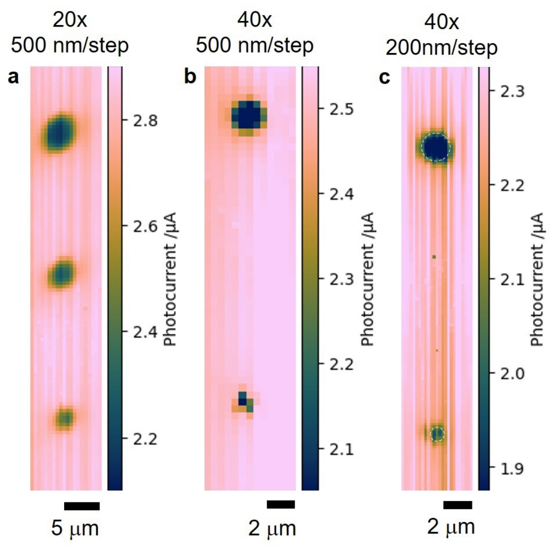

Figure S8. Raw photocurrent maps without interpolation/smoothing treatment, corresponding to **Figure 2** for (a) 5, 4 and 3 μm particles and (b) 2 and 1 μm particles. The scanning step size was 0.5 μm. (c) Raw photocurrent maps, without interpolation/smoothing treatment, obtained with 40× objective and a step size of 0.2 μm. 5 %AOTF was used.

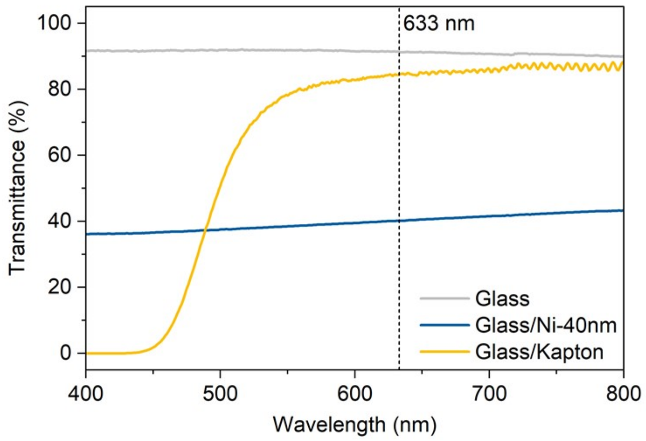

Figure S9. Transmittance spectra of a bare microscope glass slide, a microscope glass slide coated by a 40 nm-thick Ni film, and a microscope glass slide covered with Kapton tape.

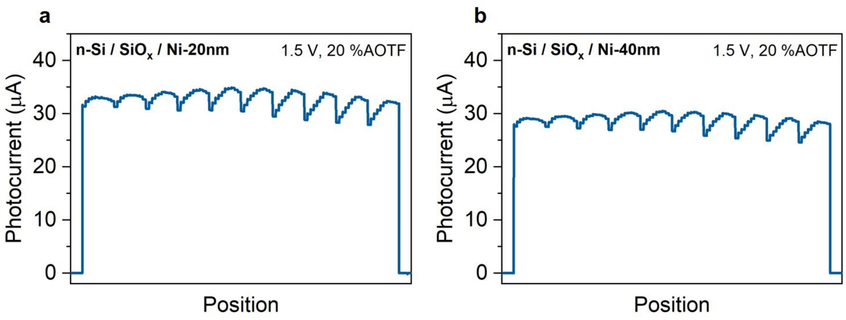

Figure S10. Photocurrent measured under local illumination on n-Si/SiO$_x$/Ni-fc photoanode covered with a Ni thickness of (a) 20 and (b) 40 nm.

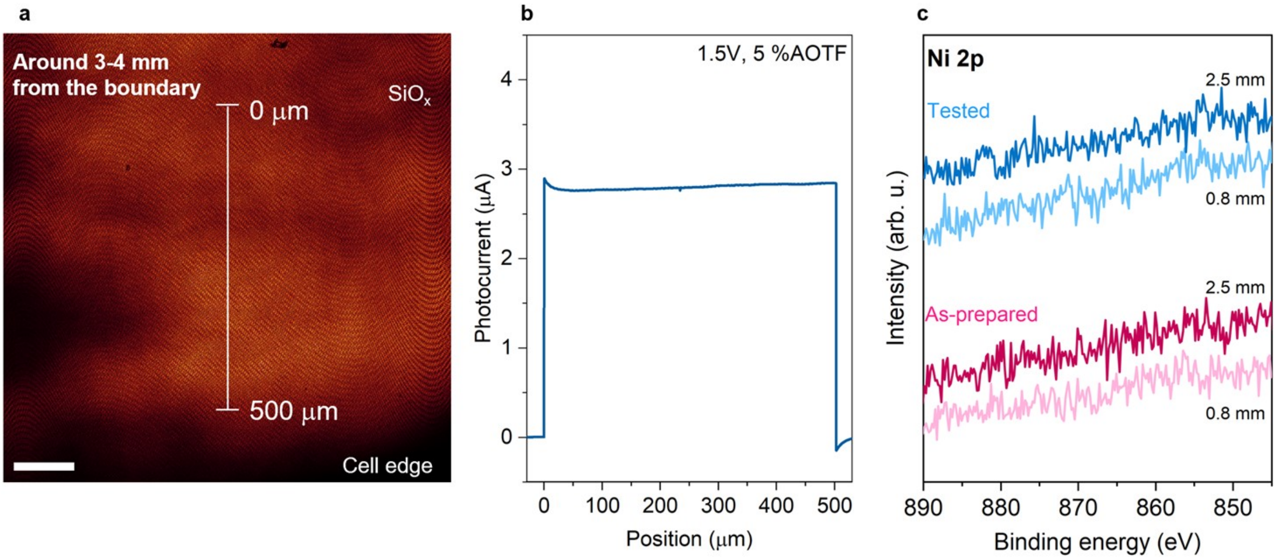

Figure S11. Photocurrent profile on n-Si/SiO$_x$/Ni-hc where the area of interest is located 3-4 mm away from the Ni boundary. (a) Confocal image of the scanned area and (b) photocurrent under local illumination following the line indicated in (a) with a scan step size of 2 μm. (c) Ni 2p XPS spectra measured on the as-prepared and tested n-Si/SiO$_x$/Ni-hc with the beam center (~400-600 μm in diameter) at various distance from the Ni boundary. Applied potential: 1.5 V, laser intensity: 5 %ATOF. The scale bar equals 100 μm.

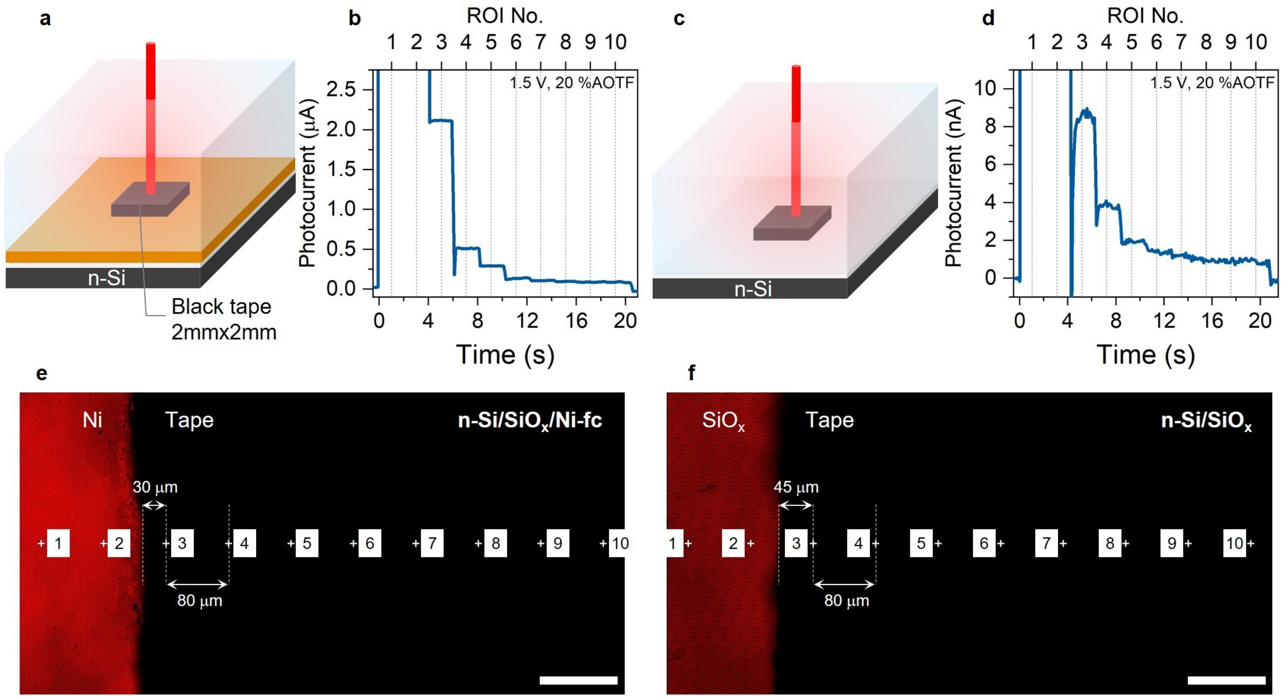

Figure S12. (a), (c) Schemes illustrating the strategy employed for measuring background photocurrents and (b), (d) measured background photocurrents on (a)-(b) *n*-Si/SiO_x/Ni-fc photoanode and (c)-(d) bare *n*-Si/SiO_x, respectively. Confocal images of (e) *n*-Si/SiO_x/Ni-fc and (f) *n*-Si/SiO_x with the small piece of the electrical tape covering the surface. The ROI/illumination positions are indicated and related to ROI no. in (b) and (d). The scale bar equals to 100 μm.

Notes on Figure S12: In these experiments, the illumination spot was located on a non-transparent electrical tape covering a part of Ni surface. Thus, there should not be any charge carrier generation and photocurrent related to the reaction. However, we observed a significant amount of photocurrent (0.5 μA), which, we believe, originates from light scattering in the electrolyte and the reflection on the surface. This is referred to as background photocurrent. The background photocurrent was much lower on bare Si, below 5 nA.

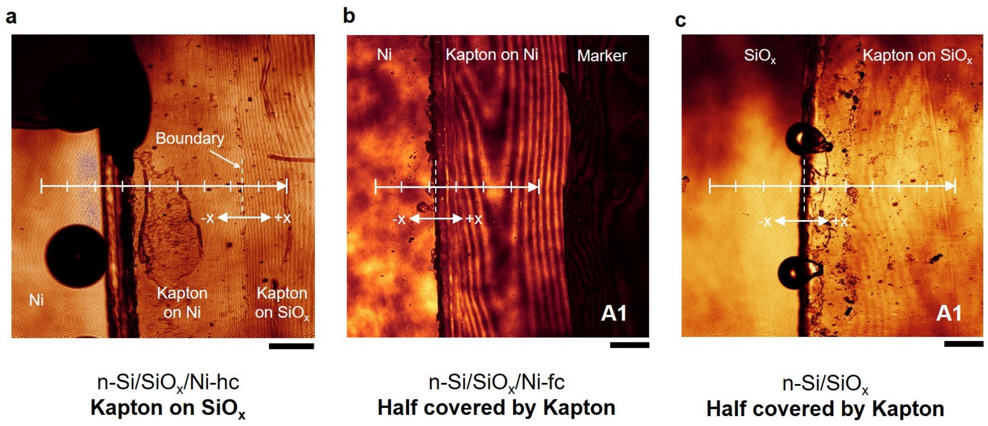

Figure S13. Confocal images and illumination point grid of the measurements for **Figure S3**: (a) *n*-Si/SiO$_x$/Ni-hc with Kapton tape on SiO$_x$; (b) *n*-Si/SiO$_x$/Ni-fc half covered by Kapton tape at A1; and (c) *n*-Si/SiO$_x$ half covered by Kapton tape at A1. The scale bar equals 100 μm. The vertical dashed lines were set as x = 0.

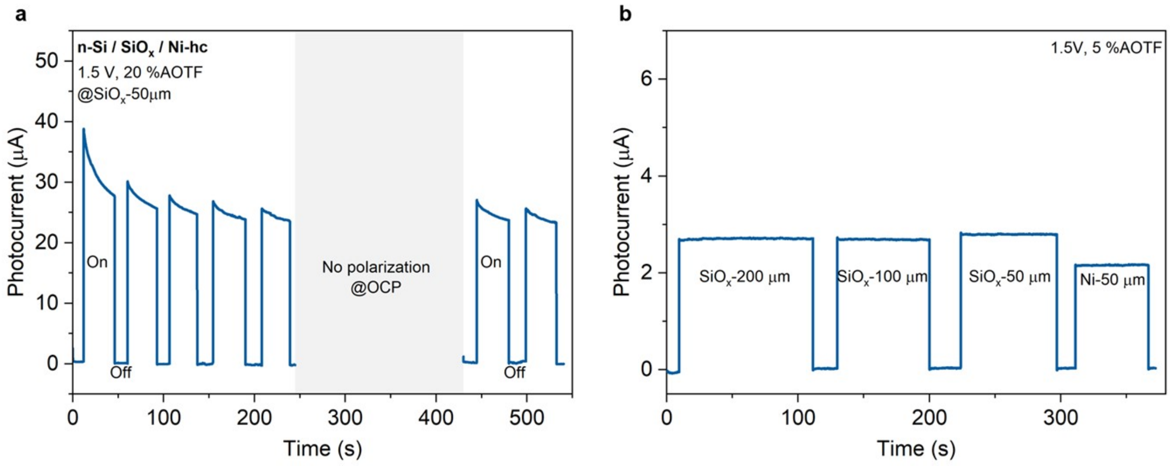

Figure S14. (a) Photocurrent measured on *n*-Si/SiO$_x$/Ni-hc under several sequential long local illumination of the SiO$_x$ surface, at 50 μm from the boundary, measured with 20 %AOTF. (b) Photocurrent measured on *n*-Si/SiO$_x$/Ni-hc under local illumination on SiO$_x$ and Ni surface at various distances from the boundary, measured with 5 %AOTF.

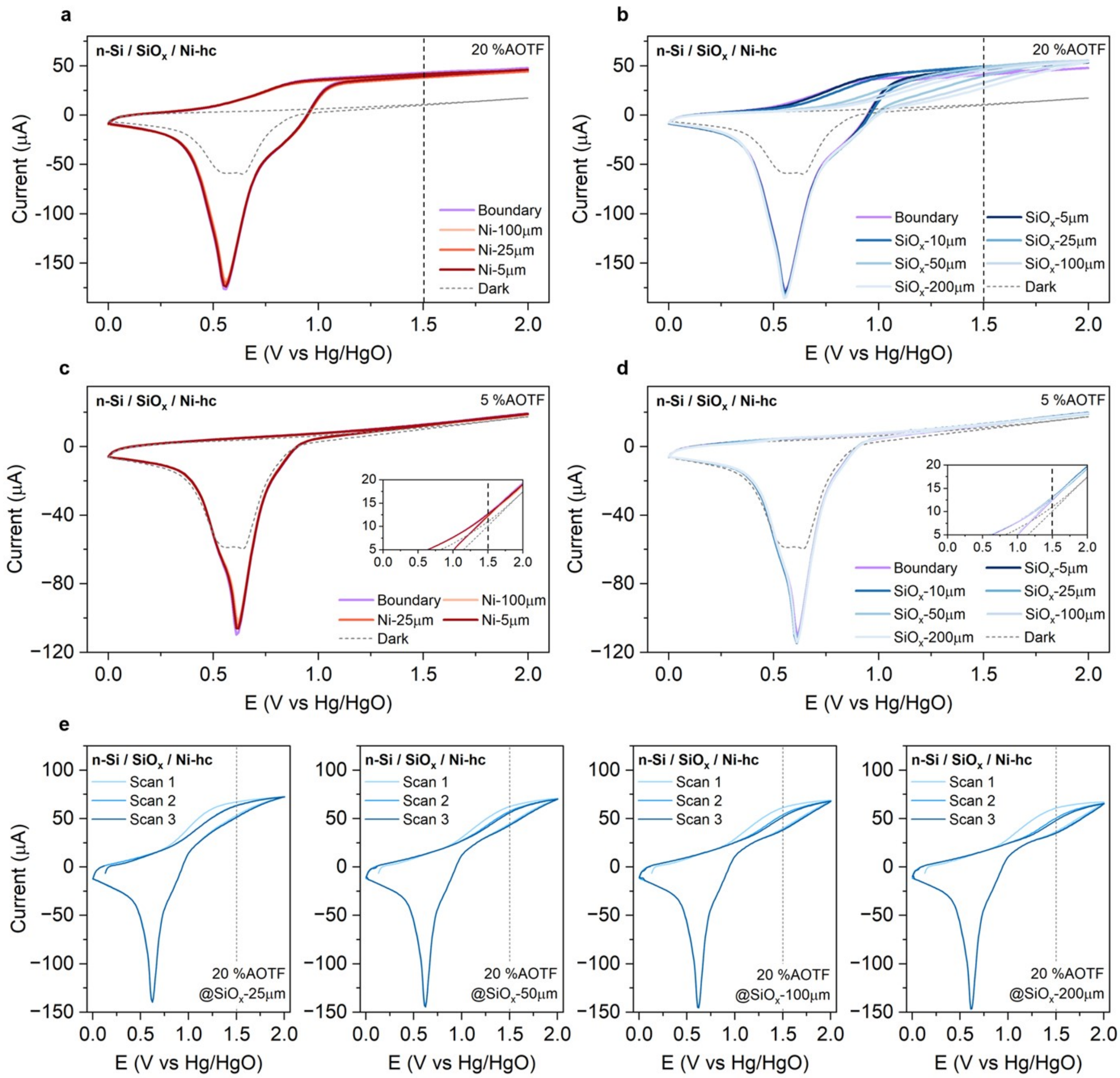

Figure S15. CVs recorded at *n*-Si/SiO_x/Ni-hc under local illumination on Ni and SiO_x at various distances from the boundary with %AOTF at (a)-(b) 20 and (c)-(d) 5. (e) CVs recorded at *n*-Si/SiO_x/Ni-hc at different scans under local illumination on SiO_x with 20 %AOTF for each distance, displaying their evolution and stabilization. Note that CV curves at 3rd scan were chosen for the comparison plot in (b). Scan rate = 100 mV s^{-1}. The dashed vertical line indicates the applied potential (1.5 V) where most photocurrent measurements were performed.

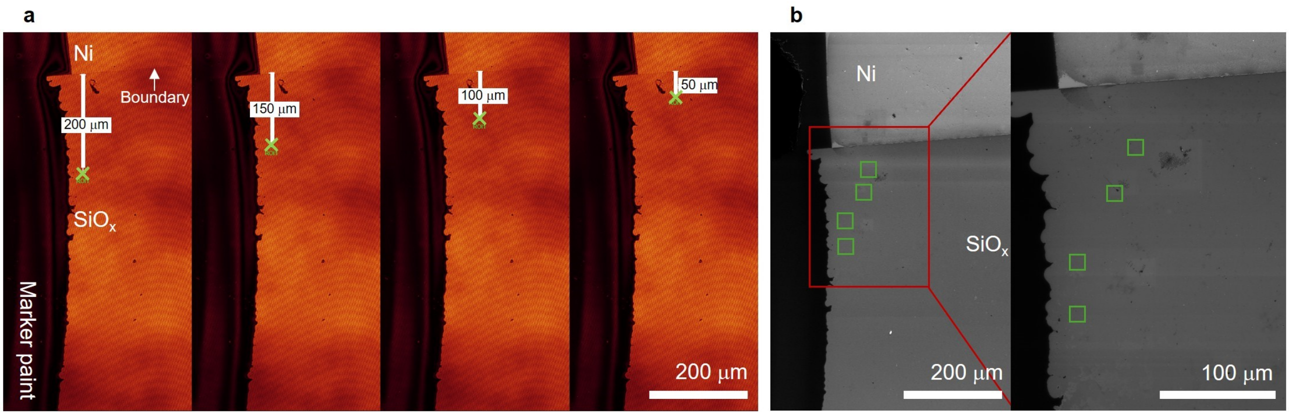

Figure S16. (a) Confocal microscopy images of the *n*-Si/SiO_x/Ni-hc photoanode displaying the local illumination points (green crosses mark the illumination points on SiO_x surface and the white line indicates the distance of the illumination point from the Ni boundary) on a photoanode at various locations. (b) SEM images corresponding to the confocal images after a long local illumination (5 minutes) at each point. Light intensity of 20 %AOTF was used in these experiments. Approximate ROIs positions are indicated by green squares in (b).

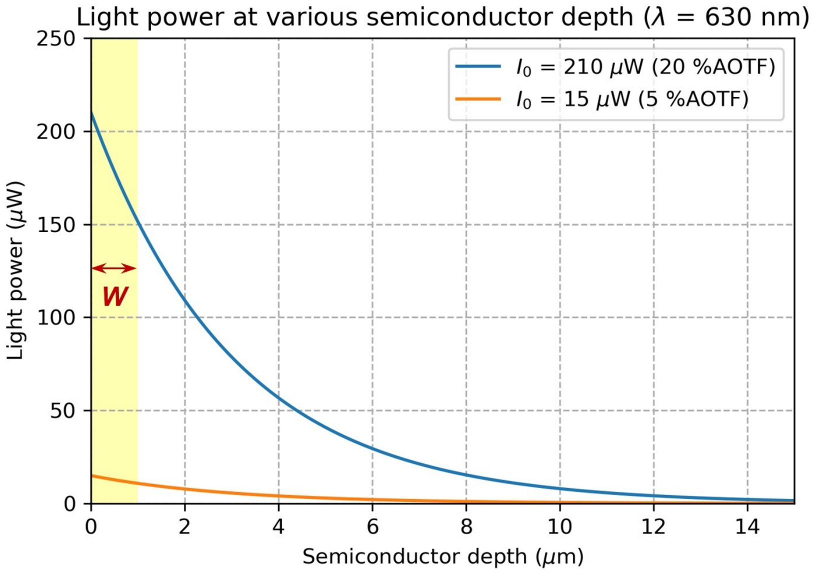

Figure S17. Simulated light power profile as a function of semiconductor/Si depth. The approximate depletion width (*W*) is shown as the yellow shaded area.

<u>Notes on Figure S17</u>: The light power profile is simulated based on the Beer-Lambert law equation as follows:[26]

$$I(z) = I_0 e^{-\alpha z} \tag{3}$$

Where $I(z)$ is the light intensity/power at a given semiconductor depth, I_0 is the initial light intensity/power, z is the semiconductor depth, and α is the absorption coefficient of Si (3.27×10^3 cm^{-1} at 630 nm).[27]

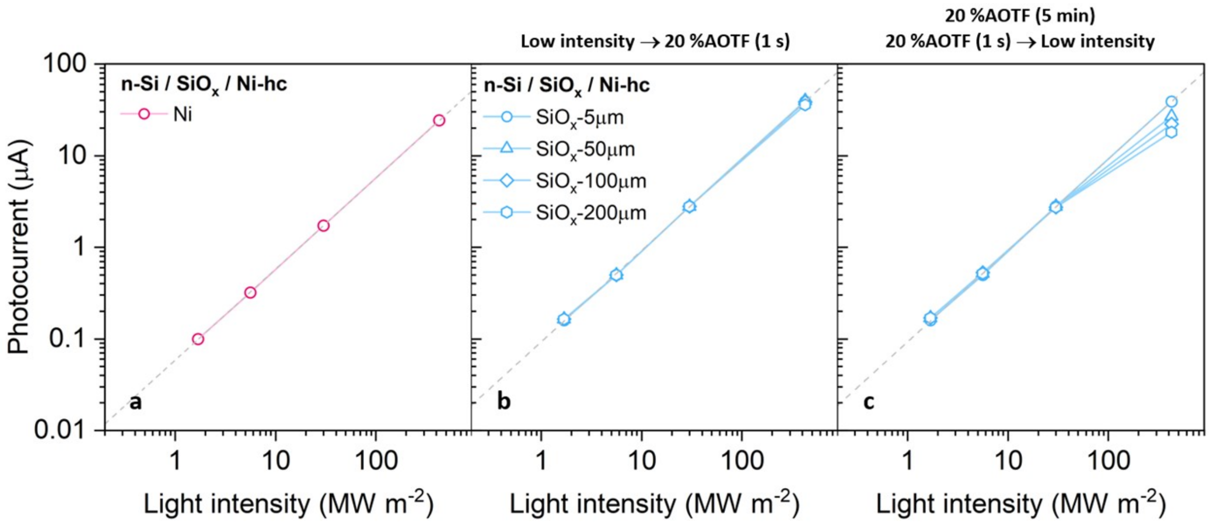

Figure S18. Photocurrent-intensity plots with the value obtained on *n*-Si/SiO_x/Ni-hc at 1.5 V under various local illumination intensity on (a) Ni and (b: fresh surface, c: modified surface by local illumination under applied bias) SiO_x region.

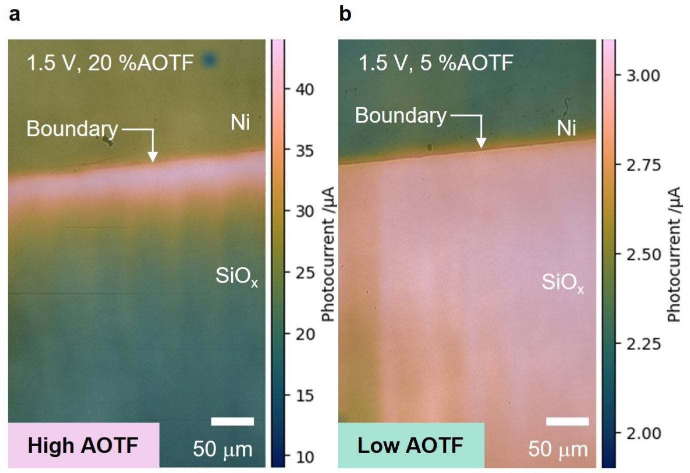

Figure S19. 2D Photocurrent maps of *n*-Si/SiO_x/Ni-hc overlaid with the confocal image obtained under (a) 20 %AOTF and (b) 5 %AOTF.

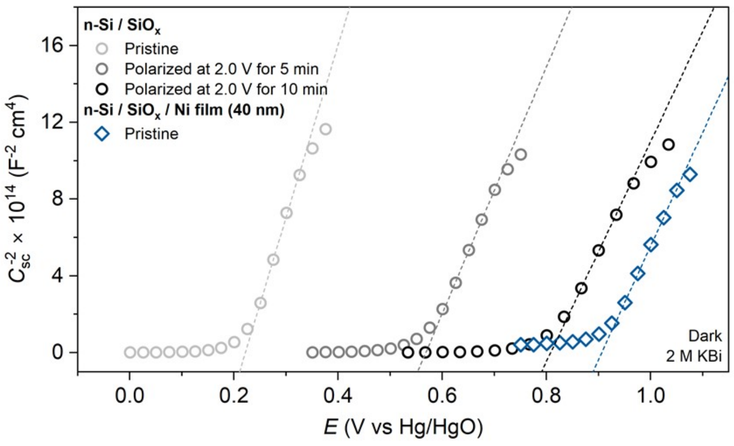

Figure S20. Mott-Schottky plots of *n*-Si/SiO_x and *n*-Si/SiO_x/Ni-fc at various conditions measured in the dark. The frequency and amplitude were set to 10 kHz and 10 mV, to obtain the C value, an equivalent circuit comprising a capacitor in parallel with a resistance was used. The electrolyte was 2 M KBi.

Notes on Figure S20:

According to the Mott-Schottky equation:[28]

$$C_{sc}^{-2} = \frac{2}{\varepsilon_0 \varepsilon_{Si} q N_D}\left(\text{Bias} - E_{fb} - \frac{kT}{q}\right) \tag{4}$$

where E_{fb} is the flat-band potential, k is Boltzmann's constant (1.38×10^{-23} m^2 kg s^{-2} K^{-1}), and T is the temperature (in K). E_{fb} can be obtained by subtracting kT/q from the x-intercept. N_D is calculated from the slope with the following equation:

$$N_D = \frac{2}{\varepsilon_0 \varepsilon_{Si} q \text{ (slope)}} \tag{5}$$

From the fitted curve, the values of the calculated N_D are relatively similar for all samples and conditions and give a resistivity of 1-2 Ω·cm, which is in good agreement with the value reported by the wafer's manufacturer (1-10 Ω·cm).

These measurements are aimed at qualitatively comparing the band bending of *n*-Si/SiO_x relative to *n*-Si/SiO_x/Ni. A lower *x*-intercept indicates a higher built-in potential and, thus, larger space charge width according to **Eq. 4**. From the Mott-Schottky plots, it can be observed that the *x*-intercept of *n*-Si/SiO_x (in all conditions) is lower than that of pristine *n*-Si/SiO_x/Ni. This reveals that the bare Si region exhibits a larger depletion layer width compared to the Ni-covered region. Nevertheless, we also found that the pristine *n*-Si/SiO_x curve shifted anodically, indicating a narrower space charge layer after the bare Si surface was polarized at 2.0 V for a certain time. Similar results have been previously reported in the literature,[11] suggesting an evolution of the *n*-Si/SiO_x junction under a continuous applied bias. We note that the liquid phase might influence the band bending at *n*-Si/SiO_x; however, as the electrolyte does not contain a well-defined redox couple to fix its potential, the determination of an absolute value of flat-band/built-in potential would be inaccurate. Conversely, for *n*-Si/SiO_x/Ni, no changes were measured

after polarization. Since the photocurrent mapping is normally carried out by applying a specific potential for a long time (>1 h), we consider the difference in depletion width formed at *n*-Si/SiO_x and *n*-Si/SiO_x/Ni to be insignificant.

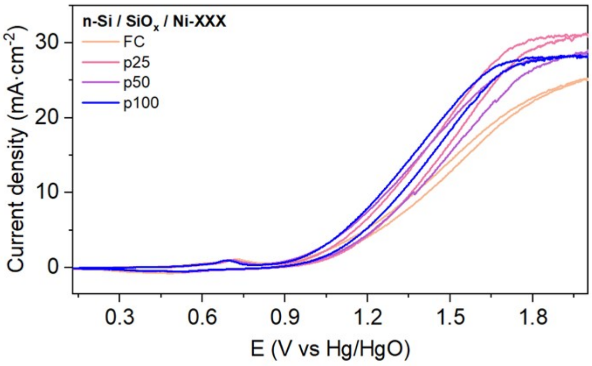

Figure S21. CVs recorded at *n*-Si/SiO_x/Ni-fc and *n*-Si/SiO_x/Ni-pΘ under 1-sun illumination in 2 M KBi.

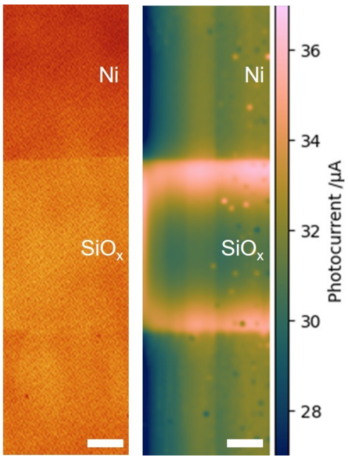

Figure S22. Confocal image and photocurrent map of a Ni pattern on *n*-Si/SiO_x/Ni-p100 obtained with an applied potential of 1.0 V and 20 %AOTF. The scan step size was 2 μm. The scale bar equals 20 μm.

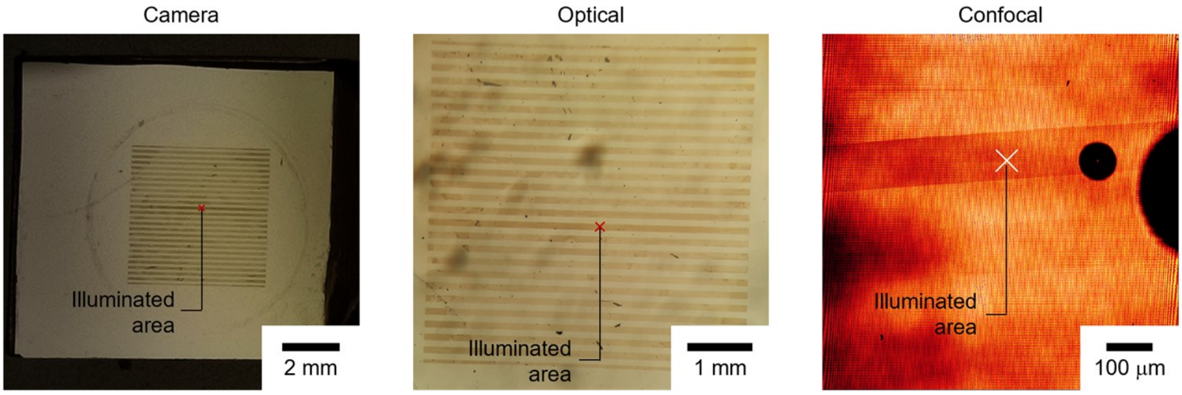

Figure S23. Images of a Ni pattern on *n*-Si/SiO_x after photocurrent mapping taken by a digital camera, optical microscope, and confocal microscope.

Section 7 - References

(1) Zhou, C.; Descamps, J.; Goudeau, B.; Garrigue, P.; Bedu, F.; Santinacci, L.; Wang, Z.; Sojic, N.; Loget, G. Photoelectrochemical Charge Transfer Imaging at Nanoscale Catalysts. *ACS Energy Lett.* **2025**, *10*, 3519–3525. https://doi.org/10.1021/acsenergylett.5c01296.

(2) Kenney, M. J.; Gong, M.; Li, Y.; Wu, J. Z.; Feng, J.; Lanza, M.; Dai, H. High-Performance Silicon Photoanodes Passivated with Ultrathin Nickel Films for Water Oxidation. *Science* **2013**, *342* (6160), 836–840. https://doi.org/10.1126/science.1241327.

(3) Song, F.; Bai, L.; Moysiadou, A.; Lee, S.; Hu, C.; Liardet, L.; Hu, X. Transition Metal Oxides as Electrocatalysts for the Oxygen Evolution Reaction in Alkaline Solutions: An Application-Inspired Renaissance. *J. Am. Chem. Soc.* **2018**, *140* (25), 7748–7759. https://doi.org/10.1021/jacs.8b04546.

(4) Zhao, Y.; Descamps, J.; Ababou-Girard, S.; Bergamini, J.; Santinacci, L.; Léger, Y.; Sojic, N.; Loget, G. Metal-Insulator-Semiconductor Anodes for Ultrastable and Site-Selective Upconversion Photoinduced Electrochemiluminescence. *Angew. Chemie Int. Ed.* **2022**, *61* (20), e202201865. https://doi.org/10.1002/anie.202201865.

(5) K. McIntosh, M. A. and B. S. *PV lighthouse*. https://www.pvlighthouse.com.au (accessed 2025-09-09).

(6) Law, M. E.; Solley, E.; Liang, M.; Burk, D. E. Self-Consistent Model of Minority-Carrier Lifetime, Diffusion Length, and Mobility. *IEEE Electron Device Lett.* **1991**, *12* (8), 401–403. https://doi.org/10.1109/55.119145.

(7) *IOFFE semiconductor database*. http://www.ioffe.ru/SVA/NSM/Semicond/ (accessed 2025-09-29).

(8) Simon M. Sze; Ng, K. K. *Physics of Semiconductor Devices*, 3rd ed.; John Wiley & Sons, 2006. https://doi.org/10.1002/0470068329.

(9) Chen, L.; Chen, M.; Lien, C.; Wan, C. The Band Model and the Etching Mechanism of Silicon in Aqueous KOH . *J. Electrochem. Soc.* **1995**, *142* (1), 170–176. https://doi.org/10.1149/1.2043860.

(10) Digdaya, I. A.; Adhyaksa, G. W. P.; Trześniewski, B. J.; Garnett, E. C.; Smith, W. A. Interfacial Engineering of Metal-Insulator-Semiconductor Junctions for Efficient and Stable Photoelectrochemical Water Oxidation. *Nat. Commun.* **2017**, *8*, 15968. https://doi.org/10.1038/ncomms15968.

(11) Molto, C.; Etcheberry, A.; Grand, P. P.; Goncalves, A. M. Study of Photo-Oxidized n-Type Textured Silicon Surface through Electrochemical Impedance Spectroscopy. *J. Electrochem. Soc.* **2020**, *167* (14), 146505. https://doi.org/10.1149/1945-7111/abc0a6.

(12) Long, K.; Kattamis, A. Z.; Cheng, I. C.; Gleskova, H.; Wagner, S.; Sturm, J. C. Stability of

Amorphous-Silicon TFTs Deposited on Clear Plastic Substrates at 250 °C to 280 °C. *IEEE Electron Device Lett.* **2006**, *27* (2), 111–113. https://doi.org/10.1109/LED.2005.863147.

(13) Jiang, C.; Moniz, S. J. A.; Wang, A.; Zhang, T.; Tang, J. Photoelectrochemical Devices for Solar Water Splitting – Materials and Challenges. *Chem. Soc. Rev.* **2017**, *46* (15), 4645–4660. https://doi.org/10.1039/C6CS00306K.

(14) Rajeshwar, K. Fundamentals of Semiconductor Electrochemistry and Photoelectrochemistry. In *Encyclopedia of Electrochemistry*; Wiley, 2002. https://doi.org/10.1002/9783527610426.bard060001.

(15) Esposito, D. V.; Levin, I.; Moffat, T. P.; Talin, A. A. H_2 Evolution at Si-Based Metal-Insulator-Semiconductor Photoelectrodes Enhanced by Inversion Channel Charge Collection and H Spillover. *Nat. Mater.* **2013**, *12* (6), 562–568. https://doi.org/10.1038/nmat3626.

(16) Digdaya, I. A.; Trześniewski, B. J.; Adhyaksa, G. W. P.; Garnett, E. C.; Smith, W. A. General Considerations for Improving Photovoltage in Metal-Insulator-Semiconductor Photoanodes. *J. Phys. Chem. C* **2018**, *122* (10), 5462–5471. https://doi.org/10.1021/acs.jpcc.7b11747.

(17) Sun, K.; Ritzert, N. L.; John, J.; Tan, H.; Hale, W. G.; Jiang, J.; Moreno-Hernandez, I.; Papadantonakis, K. M.; Moffat, T. P.; Brunschwig, B. S.; Lewis, N. S. Performance and Failure Modes of Si Anodes Patterned with Thin-Film Ni Catalyst Islands for Water Oxidation. *Sustain. Energy Fuels* **2018**, *2* (5), 983–998. https://doi.org/10.1039/c7se00583k.

(18) Descamps, J.; Zhao, Y.; Le-Pouliquen, J.; Goudeau, B.; Garrigue, P.; Tavernier, K.; Léger, Y.; Loget, G.; Sojic, N. Local Reactivity of Metal-Insulator-Semiconductor Photoanodes Imaged by Photoinduced Electrochemiluminescence Microscopy. *Chem. Commun.* **2023**, *59* (82), 12262–12265. https://doi.org/10.1039/d3cc03702a.

(19) Velazquez, J. M.; John, J.; Esposito, D. V.; Pieterick, A.; Pala, R.; Sun, G.; Zhou, X.; Huang, Z.; Ardo, S.; Soriaga, M. P.; Brunschwig, B. S.; Lewis, N. S. A Scanning Probe Investigation of the Role of Surface Motifs in the Behavior of P-WSe_2 Photocathodes. *Energy Environ. Sci.* **2016**, *9* (1), 164–175. https://doi.org/10.1039/c5ee02530c.

(20) Lee, J. Y.; Kang, S.; Lee, D.; Choi, S.; Yang, S.; Kim, K.; Kim, Y. S.; Kwon, K. C.; Choi, S. ho; Kim, S. M.; Kim, J.; Park, J.; Park, H.; Huh, W.; Kang, H. S.; Lee, S. W.; Park, H.-G.; Ko, M. J.; Cheng, H.; Han, S.; Jang, H. W.; Lee, C.-H. Boosting the Photocatalytic Hydrogen Evolution Performance via an Atomically Thin 2D Heterojunction Visualized by Scanning Photoelectrochemical Microscopy. *Nano Energy* **2019**, *65*, 104053. https://doi.org/10.1016/j.nanoen.2019.104053.

(21) Zhang, Y.; Xiao, J.; Xie, X.; Chen, H.; Deng, S. Layer Dependence of the Photoelectrochemical Performance of a WSe_2 Photocathode Characterized Using: In Situ Microscale Measurements. *RSC Adv.* **2019**, *9* (53), 30925–30931. https://doi.org/10.1039/c9ra06297a.

(22) Gelb, F.; Chueh, Y.-C.; Sojic, N.; Keller, V.; Zigah, D.; Cottineau, T. Electrosynthesis of

Gradient TiO_2 Nanotubes and Rapid Screening Using Scanning Photoelectrochemical Microscopy. *Sustain. Energy Fuels* **2020**, *4* (3), 1099–1104. https://doi.org/10.1039/C9SE00895K.

(23) Zhu, H.; Xie, H.; Yang, Y.; Wang, K.; Zhao, F.; Ye, W.; Ni, W. Mapping Hot Electron Response of Individual Gold Nanocrystals on a TiO_2 Photoanode. *Nano Lett.* **2020**, *20* (4), 2423–2431. https://doi.org/10.1021/acs.nanolett.9b05125.

(24) Wang, Y.; Chen, C.; Zhu, P.; Shen, J.; Zou, S.; Dong, W.; Su, X.; Ni, W.; Fan, R.; Shen, M. Enhanced Solar Hydrogen Production by Manipulating the Spatial Distribution of Pt Catalyst onto Si Micro-Pyramid Arrays. *Appl. Phys. Lett.* **2023**, *122* (19), 193902. https://doi.org/10.1063/5.0141701.

(25) Geng, T.; Wang, L.; Hu, N.; Li, H.; Zhang, H.; Feng, X.; Li, L.; Jiang, J.-H.; Liz-Marzán, L. M.; Ni, W. Mapping Plasmon-Enhanced Photocurrent in Single Au Nanoplate/MoS_2 Heterostructures. *ACS Photonics* **2025**, *12* (7), 3735–3744. https://doi.org/10.1021/acsphotonics.5c00701.

(26) Mayerhöfer, T. G.; Pahlow, S.; Popp, J. The Bouguer-Beer-Lambert Law: Shining Light on the Obscure. *Chemphyschem* **2020**, *21* (18), 2029–2046. https://doi.org/10.1002/cphc.202000464.

(27) Green, M. A.; Keevers, M. J. Optical Properties of Intrinsic Silicon at 300 K. *Prog. Photovoltaics Res. Appl.* **1995**, *3* (3), 189–192. https://doi.org/10.1002/pip.4670030303.

(28) Sivula, K. Mott-Schottky Analysis of Photoelectrodes: Sanity Checks Are Needed. *ACS Energy Lett.* **2021**, *6* (7), 2549–2551. https://doi.org/10.1021/acsenergylett.1c01245.
